# Sample-Conditional Mixtures of Entropy Estimators for Short Sequences Under a Uniform Marginal Null

**DOI:** 10.3390/e28101073

**Published:** 2026-09-30

**Authors:** Guillermo Sosa-Gómez

**Affiliations:** 1 Facultad de Ciencias Económicas y Empresariales, Universidad Panamericana, Álvaro del Portillo 49, Zapopan 45010, Jalisco, Mexico; gsosag@up.edu.mx; 2 Laboratory for Reputation Management in Education, HSE University Saint-Petersburg, Griboedova Embankment 123, Office 502, Saint Petersburg 190068, Russia

**Keywords:** entropy estimation, cryptography, short samples, adaptive mixture, James–Stein shrinkage, pseudorandom number generators, frequency-of-frequencies

## Abstract

Entropy estimation from short samples recurs in symmetric cryptography, where the reference distribution is uniform by design and no single estimator in the considered classical comparison set minimizes mean squared error (MSE) across the full range of ratios n/k. We introduce the adaptive sample-conditional entropy diagnostic (ASED), in which a compact network trained offline maps a frequency-of-frequencies descriptor of the sample to convex mixture weights over six classical estimators, at a cost of O(n+k). We prove an oracle inequality bounding the excess risk of such a mixture by the $L_{1}$ error of its weights and fixed-alphabet consistency for every simplex-valued weighting rule, independently of the distribution used to train the weights. Under the uniform-null protocol, ASED attains an integrated MSE of $1.7\times 10^{-3}$ for bytes, compared with $3.8\times 10^{-2}$ for James–Stein shrinkage, a reduction that depends materially on the aggregation scheme: 95.4% for summed or averaged MSE across sample sizes versus 24% for the mean of per-size ratios. Both figures are reported together throughout, and neither is presented as the headline; the entire advantage is confined to the undersampled regime n<k, the two estimators being indistinguishable for n≥k. A locked train/validate/test evaluation with an independently written implementation reproduces the reduction at 95.1%. Because a constant output achieves zero error by construction here, we add further checks: in a post hoc analysis, ASED is non-inferior to SHR in detection power at a 0.02 margin, both pointwise and simultaneously, whereas a constant control has none, and the advantage persists on held-out configurations. The 0.02 margin was selected after inspecting the power estimates, and at n=8 and n=16, it is smaller than the variation induced by tie handling at the empirical critical value, so the non-inferiority conclusion is confirmatory only for n≥32. At n≤16, the two statistics have identical null distributions up to monotone relabeling, and no test of size 0.05 exists, so no power difference is identifiable in either direction, and the comparison is withdrawn there. For strongly non-uniform sources, the ordering reverses, and NSB dominates, delimiting ASED as a uniformity diagnostic rather than a general-purpose entropy estimator. The design choices behind ASED, the feature map, architecture, and loss weighting were made while observing results on this same evaluation protocol, so no independent model-selection split separates development from the assessment reported here. That pipeline is accordingly designated exploratory, and the locked evaluation is confirmatory. The contribution is stated as sample-conditional convex aggregation of classical estimators rather than as the particular network realizing it: a five-feature variant and a gradient-boosted surrogate perform comparably, with no paired interval separating the three. Claims are restricted to short i.i.d. samples and marginal Shannon-entropy diagnostics under a uniform null; min-entropy, unpredictability, dependence, and randomness certification are out of scope.

## 1. Introduction

Shannon entropy measures the uncertainty of a discrete information source through the distribution of its output symbols [1]. In symmetric cryptography, it plays a dual role. At design time, entropy is a quantitative criterion: candidate S-boxes, filtering functions, and generator configurations are compared, in part, by how close the empirical entropy of their outputs lies to the theoretical maximum $H_{\max}=\log_{2}k$ attained by the uniform distribution over an alphabet of size *k*. At evaluation time, entropy estimation underpins statistical batteries and health tests: a PRNG, a stream cipher keystream, or the output of an S-box construction are expected to be indistinguishable from uniform, so any systematic deviation of the estimated entropy from $H_{\max}$ signals a detectable bias with potential security consequences [2,3].

The samples available for these judgments are often short, far shorter than the asymptotic regime in which classical estimators behave well. Standardized test suites expose short-window behavior: NIST SP 800-22 [4] and TestU01 [5] include statistics computed over local block structures or subwindows, while SP 800-90B [6] includes restart and health-test components for which short-window behavior is directly relevant. Design loops aggravate the problem: evolutionary and heuristic S-box search procedures evaluate thousands of candidate constructions per run, so the per-candidate sample budget is necessarily small [7], and initialization-phase analysis of stream ciphers examines the first few hundred output symbols [3] precisely. Diagnosing marginal biases that manifest only over short windows, a biased initialization vector expansion, and a transient change in symbol frequencies require estimators that remain reliable when *n* is comparable to or smaller than the alphabet size. Temporal-dependence failures, such as a transient correlation after reseeding, fall outside this scope: they may leave the marginal distribution exactly uniform and are the province of dependence, autocorrelation, or conditional-entropy tests, as Section 4.10 illustrates.

Estimating entropy from short samples is intrinsically difficult. No unbiased estimator exists [8], and the convergence rate of any consistent estimator can be made arbitrarily slow [9]. A sample of size Ω(k/logk) is necessary for consistent estimation at fixed precision [10], a bound routinely violated in the block-level settings above. In the undersample regime where n<k, the maximum-likelihood plug-in estimator exhibits severe downward bias, recovering only a fraction of the true entropy [11]. Substantial literature responds with correction terms [12,13,14], Bayesian priors [15,16,17,18], coverage-based methods [10,19,20,21], and empirical Bayes shrinkage [22]. The most comprehensive comparison for the cryptographic setting is due to Contreras Rodríguez et al. [2], who evaluated a broad battery of classical estimators over byte and bit sequences from n=8 to n=16,384 and found the James–Stein shrinkage estimator (SHR) to be the most effective single estimator for byte samples.

That comparison, however, also exposed a structural difficulty: the identity of the best estimator depends on the regime ratio n/k. Bayesian smoothers dominate deep in the undersample regime, shrinkage dominates in an intermediate band, and bias-corrected plug-in estimators take over as n/k grows. A practitioner must nevertheless commit to a single estimator family before seeing the data, even though some members tune their parameters from the sample, and a mismatch between the chosen estimator and the actual regime results in avoidable bias and inflated variance. No estimator in the comparison adaptively combines heterogeneous estimator families using sample-level occupancy information: several tune their own internal parameters from the data; SHR selects its shrinkage intensity; NSB integrates a data-dependent posterior; and coverage-based estimators use sample statistics, but each remains a single family. ASED introduces this second-level adaptivity by mapping the observed occupancy profile to mixture weights, as the regime-dependence of the ranking calls for.

A scope clarification is necessary before describing our proposal, because the word “entropy” covers two distinct quantities in cryptographic practice. Security certification of entropy sources is the business of SP 800-90B [6], and AIS 20/31 [23] is formulated in terms of min-entropy $H_{\infty }=-\log_{2}\max_{x}p_{x}$, which lower-bounds the work of an optimal guessing adversary and is deliberately more conservative than Shannon entropy. The present paper does not address min-entropy estimation. Its setting is the complementary one in which Shannon entropy is the established figure of merit: comparative design diagnostics and uniformity testing of PRNG, S-box, and keystream outputs, where the null hypothesis is the uniform distribution and the quantity of interest is the gap $H_{\max}-H$ [2,3]. Conclusions drawn here transfer to certification pipelines only insofar as Shannon-entropy diagnostics complement, rather than replace, min-entropy estimators. A second restriction belongs here rather than in the results, because it bounds everything that follows: ASED is trained against the uniform null and is a uniformity-oriented Shannon-entropy diagnostic, not a general-purpose entropy estimator. On strongly skewed (Zipf s=1) or periodic sources, it is outperformed by NSB and by SHR, in places by more than an order of magnitude, and Section 4.9 quantifies that boundary rather than eliding it. No claim in this paper extends beyond near-uniform i.i.d. sources over the sample-size grid evaluated here, and the method should not be used as a screening tool on sources whose marginal is expected to be far from uniform.

This paper proposes the adaptive sample-conditional entropy diagnostic (ASED), an estimator that addresses the regime-commitment problem by learning a convex mixture of base estimators whose weights are conditioned on a compact occupancy-profile representation of the observed sample. The representation is derived from the frequency-of-frequencies (FoF) histogram, a vector that counts how many symbols were observed exactly *i* times, capturing the macroscopic regime (n/k, coverage, singleton density) without reference to symbol identities. A lightweight multilayer perceptron (MLP), trained once offline on synthetic data, maps this representation to weights on the probability simplex over six complementary base estimators, so that adaptive estimation costs no more than a constant number of plug-in evaluations.

Designing the evaluation of such an estimator requires care on one methodological point, which we flag from the outset. ASED is trained against the uniform null, and under a purely uniform-null evaluation protocol, the constant “estimator” $\hat{H}\equiv \log_{2}k$ achieves zero error by construction. Low MSE under the null is therefore a necessary but not sufficient condition for usefulness: a meaningful adaptive estimator must, in addition, retain sensitivity to departures from uniformity. We therefore treat the uniform-null comparison as only the first of three evidentiary layers, and complete it with (i) a power analysis against near-uniform alternatives of known entropy, in which a constant control exhibits the confound explicitly and (ii) generalization tests on held-out sample sizes and an unseen alphabet.

Our contributions are threefold. First, we formalize the occupancy-profile regime descriptor derived from the FoF histogram, prove that every label-invariant plug-in estimator is a function of the FoF vector, and define the resulting 37-dimensional feature map (Section 3). Second, we introduce the ASED architecture: frequency-of-frequencies feature extraction, offline training of the weight mapper on synthetic data, and adaptive convex combination at inference, with O(n+k) inference complexity. For it we prove three results: an oracle inequality bounding its excess risk over the sample-conditional oracle by the $L_{1}$ approximation error of the learned weights; a checkable covariance condition under which no regime-indexed fixed convex mixture of the specified base estimators can improve on its best member whenever this condition holds, sample-dependent weighting is necessary for improvement within that mixture class; and a distribution-free bound under which ASED inherits the consistency and convergence rate of its base set whatever the training distribution, sharpened under a checkable hypothesis into a bound relative to the conditionally best estimator (Section 3). Appendix B adds an idealized PAC bound, kept out of the main development because its numerical value is vacuous at our training size. Third, we evaluate ASED in three layers: the uniform-null protocol of Contreras Rodríguez et al. [2] with 1000 replications and bootstrap uncertainty, a power analysis against near-uniform alternatives with the constant estimator as negative control, and generalization tests on held-out configurations, obtaining a reduction in integrated MSE over SHR for byte sequences of 95.4% under summed or averaged aggregation and 24% under the mean of per-size ratios two figures reported together throughout, since the headline depends materially on which is used (Section 4.4) with detection power non-inferior to SHR’s at a post hoc 0.02 margin, confirmatory for n≥32 and exploratory at n=8 and n=16 (Section 4.10.2), (Section 4 and Section 5).

The remainder of the paper is organized as follows. Section 2 reviews the entropy estimation literature relevant to the short-sample cryptographic setting. Section 3 presents the ASED model. Section 4 reports the experimental evaluation. Section 5 discusses scope, limitations, and practical implications, and Section 6 concludes.

## 2. Literature Review

### 2.1. Entropy Estimation: Foundations

The Shannon entropy of a discrete random variable *X* with alphabet K and probability function $p_{x}=\mathrm{Pr}\left\{X=x\right\}$ is

(1) $$H\left(X\right)=-\sum_{x\in K}p_{x}\log_{2}p_{x},$$

reaching its maximum $H_{\max}=\log_{2}\left|K\right|$ under the uniform distribution [1]. Two statistical constraints fundamentally limit estimation: no unbiased estimator of H(X) exists [8], and any consistent estimator may converge arbitrarily slowly [9]. A sample of size Ω(k/logk) is necessary for consistent estimation at any fixed precision [10], a bound routinely violated in cryptographic practice.

The maximum-likelihood (ML) plug-in estimator ${\hat{H}}_{\mathrm{ML}}=-\sum_{x}{\hat{p}}_{x}^{\;\mathrm{ML}}\log_{2}{\hat{p}}_{x}^{\;\mathrm{ML}}$, where ${\hat{p}}_{x}^{\;\mathrm{ML}}=y_{x}/n$ is the empirical frequency, consistently underestimates *H* for n<k. Miller [12] proposed the correction ${\hat{H}}_{\mathrm{MM}}={\hat{H}}_{\mathrm{ML}}+\left(m_{\mathrm{obs}}-1\right)/\left(2n\ln2\right)$ (in bits; the classical form is in nats), where $m_{\mathrm{obs}}$ counts observed symbols, yielding faster convergence but residual bias for small n/k. Paninski [8] derived the best upper bound (BUB) estimator via polynomial approximation, proving dominance over ML, MM, and Jackknife in neural spike-train settings.

### 2.2. Bayesian and Empirical Bayes Estimators

Bayesian estimators replace ML frequencies with posterior expectations under Dirichlet priors parameterized by a>0: common choices include the Jeffrey prior (a=1/2, Krichevsky and Trofimov [15]), Laplace smoothing (a=1, Holste et al. [16]), the Schürmann–Grassberger prior (a=1/k, Schürmann and Grassberger [24]), and the minimax prior ($a=\frac{\sqrt{n}}{k}$, Trybula [17]). The NSB estimator Nemenman et al. [18] integrates over a calibrated prior, achieving strong performance for general distributions.

Hausser and Strimmer [22] showed that SHR, which shrinks ML frequencies toward the uniform distribution, is an empirical Bayes estimator that selects its shrinkage intensity from the data. This self-tuning property gives SHR a clear advantage in the undersample regime and identifies it as the best single estimator for byte sequences in Contreras Rodríguez et al. [2].

### 2.3. Coverage-Based Methods

The Chao–Shen (CS) estimator [19] combines Good–Turing coverage correction with Horvitz–Thompson weighting to account for unseen symbols. Its coverage estimate $C=1-f_{1}/n$, where $f_{1}$ is the singleton count, is a key element of the FoF representation that forms the core of our occupancy-profile regime descriptor. The Chao–Wang–Jost (CWJ) estimator [20] extends the species-accumulation framework, while the Unseen estimator [10] is designed for the general non-uniform undersample regime.

### 2.4. Mixture, Ensemble, and Adaptive Estimation

Combining predictors is a classical idea: stacked generalization [25], stacked regressions [26], and the super learner framework with its oracle guarantees [27] all construct weighted combinations of base procedures. Entropy estimation already contains an implicit form of model averaging: the NSB estimator [18,28] integrates the Dirichlet family of Bayesian estimators against a hyperprior, which can be read as a continuous mixture over the parameter *a* with data-dependent weights. ASED can be viewed as a discrete, learned counterpart to this construction: instead of averaging over a single parametric family under a fixed hyperprior, it combines six heterogeneous estimators with weights produced by a function fitted offline. To the best of our knowledge, a learned convex mixture of heterogeneous entropy estimators, conditioned on sample statistics, has not been studied in the short-sample cryptographic setting. The key obstacle for classical stacking in this setting is the absence of a validation set: with a single observed sample, cross-validation would consume the data needed for estimation. ASED sidesteps the obstacle by fitting the mixing function offline on synthetic data, with the scope implications discussed in Section 3 and Section 5.

Weighting several procedures by a learned, covariate-dependent gate is also the defining structure of mixtures of experts [29,30] and of feature-weighted stacking [31], and the statistical theory of aggregation supplies rates for combining a finite dictionary of procedures [32,33]. ASED is an instance of that structure, and we do not claim that conditional mixing is new; three features distinguish the present setting. The gate reads a label-invariant summary of the same sample the base procedures consume, rather than an exogenous covariate, so weights and base estimates are statistically dependent, and aggregation results that assume independent weights or sample splitting do not transfer directly. The dictionary consists of estimators of a single functional rather than predictors of an observable response, so there is no held-out label against which to fit the gate; hence, we substitute offline synthetic supervision with a known target. Furthermore, the operating regime is a single short sample, where cross-validated stacking has nothing to split. The novelty claim is accordingly narrow: learned sample-conditional convex aggregation for short-sample entropy estimation under a uniform marginal null.

### 2.5. Shannon Entropy, Min-Entropy, and Cryptographic Practice

Operational standards distinguish two figures of merit. Certification of physical and non-physical entropy sources under SP 800-90B [6] and AIS 20/31 [23] is based on min-entropy $H_{\infty }=-\log_{2}\max_{x}p_{x}$, estimated by dedicated procedures (most-common-value, collision, Markov, and compression estimators), because $H_{\infty }$ bounds the success probability of an optimal guessing adversary and is the conservative choice for security claims. Shannon entropy, by contrast, is the established figure of merit for comparative design diagnostics and uniformity analysis: NIST SP 800-22 [4] and TestU01 [5] evaluate statistics on short blocks, entropy appears among the information-theoretic criteria of S-box design evaluation [7], and keystream analyses quantify the gap to $H_{\max}$ over initial output windows [3]. ASED targets this second, Shannon-entropy setting; it is not a min-entropy estimator and makes no certification claims.

## 3. The ASED Model

### 3.1. Occupancy-Profile Feature Extraction

Let $x=(x_{1},\ldots ,x_{n})$ be an observed sample of size *n* drawn from alphabet K of size *k*. Denote $y_{x}=\left|\left\{i:x_{i}=x\right\}\right|$ the count of symbol *x*. The frequency-of-frequencies (FoF) sequence is

(2) $$f_{i}\;=\;\left|\{x\in K:y_{x}=i\}\right|,\;i=0,1,\ldots ,n.$$

**Lemma 1**  (Representation)**.** *Every plug-in entropy estimator that is invariant under relabeling of the alphabet, i.e., that depends on the data only through the multiset of counts $\left\{y_{x}:x\in K\right\}$, is a function of the FoF vector $\left(f_{0},f_{1},\ldots ,f_{n}\right)$.*

**Proof.**  A label-invariant estimator is a symmetric function of the multiset $\left\{y_{x}:x\in K\right\}$, and a multiset of non-negative integers is fully characterized by its empirical distribution $\left(f_{0},\ldots ,f_{n}\right)$.    □

All six base estimators used below are label-invariant, so by Lemma 1, the complete FoF vector $\left(f_{0},\ldots ,f_{n}\right)$ is a lossless representation for the purpose of combining them. The 37-dimensional descriptor of Equation (3) is a truncated compression of that vector: it retains $f_{1},\ldots ,f_{34}$ and discards $f_{i}$ for i≥35, so for n>34, it does not determine that the base estimates ${\hat{H}}_{\mathrm{ML}}=-\sum_{i}f_{i}\left(i/n\right)\log_{2}\left(i/n\right)$ already depend on the discarded coordinates, and it is not information-preserving in general. Its adequacy is an empirical matter, supported by the truncation and ablation experiments of Section 4.7 and Section 4.9; the theorems below are unaffected, since they are stated conditionally on the descriptor φ actually used. (We do not claim statistical sufficiency of the FoF vector in the Fisherian sense; Lemma 1 is the purely combinatorial statement needed here.) We define the occupancy-profile feature vector $\varphi \in R^{37}$ as

(3) $$\varphi \;=\;\left[\underset{\mathrm{regime}\;\mathrm{summary}}{\underset{︸}{n/k,\;C,\;f_{1}/n,\;f_{2}/n,\;\hat{s}}},\;\underset{\mathrm{FoF}\;\mathrm{tail}}{\underset{︸}{f_{3}/n,\;f_{4}/n,\;\ldots ,\;f_{34}/n}}\right],$$

where $C=1-f_{1}/n$ is the Good–Turing coverage estimate, and $\hat{s}=\sum_{x}1\left[y_{x}>0\right]/k$ is the fraction of the alphabet observed. The first five entries encode the macroscopic regime: n/k captures the fundamental undersample ratio; *C* and $f_{1}/n$ encode singleton density; $f_{2}/n$ appears in second-order coverage corrections; and $\hat{s}$ reflects alphabet coverage. The trailing 32 entries $f_{3}/n,\ldots ,f_{34}/n$ capture higher-order regularity of the sample. Note that *C* and $f_{1}/n$ are affinely dependent by construction ($C=1-f_{1}/n$), so the map carries 36 functionally independent coordinates; we retain both because the redundant parameterization is harmless for a standardized linear input layer and keeps the coverage and singleton readings explicit. Section 4.7 shows that the first five coordinates retain all measurable performance in the i.i.d. uniform and near-uniform settings evaluated here, so a practitioner may use the five-feature variant (“ASED-lite”) without measurable loss under this experimental protocol. The feature vector is computable in O(n+k) time.

### 3.2. Weight Mapper Architecture

The weight mapper $\phi_{\theta }:R^{37}\to \Delta^{M}$ is an MLP mapping occupancy-profile features to the probability simplex over M=6 base estimators:

(4) $$\phi_{\theta }\left(\varphi \right)\;=\;\mathrm{softmax}\;\left(W_{3}\;\sigma \;\left(W_{2}\;\sigma \left(W_{1}z+b_{1}\right)+b_{2}\right)+b_{3}\right),\;z=\left(\varphi -\mu \right)⊘\varsigma ,$$

with hidden dimension 64, activation σ=ReLU, feature standardization by the training mean μ and standard deviation ς, and a softmax output ensuring $\sum_{j}\lambda_{j}=1$, $\lambda_{j}\geq 0$.

#### Base Estimator Set

The six base estimators in B are selected for complementary strengths across regimes:

- SHR: empirical Bayes shrinkage toward uniform [22]; the strongest single estimator at small n/k.
- MM: Miller–Madow correction [12]; asymptotically efficient for n≫k.
- CS: Chao–Shen coverage correction [19]; robust to unseen symbols.
- LAP: Laplace smoothing (a=1) [16]; low variance at very small *n*.
- MIN: minimax Dirichlet prior ($a=\frac{\sqrt{n}}{k}$) [17]; strong for tiny alphabets.
- SG: Schürmann–Grassberger prior (a=1/k) [24]; near-ML behavior with mild regularization.

Two earlier candidates for the base set were excluded for reproducibility reasons: BUB [8] requires solving a regularized polynomial-approximation problem, and the centered Dirichlet mixture requires numerical hyperprior integration, so neither admits the closed-form, dependency-free reference implementation this paper commits to; MIN and SG were included instead as closed-form Dirichlet members covering, respectively, small-alphabet strength and near-ML behavior.

Total weight-matrix parameter count: 37×64+64×64+64×6=6848 (plus 64+64+6=134 biases). Table 1 consolidates all architecture and training hyperparameters. The reference implementation of ASED and of the six base estimators is released as Supplementary Materials (Code S1), and the trained weight-mapper coefficients together with the corresponding feature standardization constants μ and ς are released as Supplementary Materials (Data S1).

An immediate structural property is worth recording: because the output of ASED is a convex combination of the base estimates, it always lies in the interval $\left[\min_{j}{\hat{H}}_{j},\;\max_{j}{\hat{H}}_{j}\right]$. Convexity therefore anchors the output to the range of the base estimators, but it does not by itself rule out a constant-valued adaptive combination: whenever $H_{\max}$ lies in that interval, a sample-dependent λ(φ) could return $H_{\max}$ for every sample. Whether the fitted model behaves that way is an empirical question, addressed by the power experiments of Section 4.5.

### 3.3. Offline Training

The weight mapper is trained on synthetic samples drawn from Uniform(K) for k∈{2,256} across the same 12 sample sizes used in evaluation. For each configuration (k,n), we generate 1000 samples, extract φ, evaluate all base estimators, and minimize a *regime-balanced* squared error with an annealed entropy bonus:

(5) $$L\left(\theta \right)\;=\;E_{n,k}\;\left[w\left(k,n\right){\left(\sum_{j=1}^{M}\lambda_{j}\left(\varphi ;\theta \right)\;{\hat{H}}_{j}\;-\;H_{\max}\right)}^{\;2}\right]\;-\;\beta_{t}\;E\left[H(\lambda )\right],$$

where $w\left(k,n\right)\propto 1/\min_{j}E\left[{\left({\hat{H}}_{j}-H_{\max}\right)}^{2}\right]$ equalizes the contribution of each (k,n) regime, H(λ) is the Shannon entropy of the weight vector, and $\beta_{t}$ anneals linearly from 1 to 0 over the first 30% of training. Both terms address a concrete failure mode: under the unweighted loss, the softmax saturates early on the globally strong SHR corner of the simplex and never recovers the large relative improvements available at the smallest sample sizes. Optimization uses Adam with learning rate $10^{-3}$, cosine annealing over 600 epochs, and batch size 256; training takes under one minute on a single CPU core. Across three independent initialization and shuffling seeds, the resulting integrated MSE on a common evaluation set agrees to three significant figures, so the balanced objective also removes seed sensitivity. No sample used for training is reused during evaluation, eliminating sample-level leakage; configuration-level generalization is tested directly in Section 4.6. We state one methodological caveat plainly. The design choices for the feature map, the tail length, the hidden widths, the regime weighting, and the entropy bonus were made while inspecting results on the same evaluation protocol, so no independent model-selection split separates development from final assessment. Sample-level leakage is excluded, but experimenter-level overfitting is not, and a locked-test protocol (train/validate/final test) would be required to exclude it. The held-out configurations of Section 4.6 and the alternative sources of Section 4.9 and Section 4.10, which were never used for tuning, are the closest substitutes available here. Because a model-selection split cannot be imposed retrospectively, we state explicitly how the two bodies of evidence in this paper relate to one another, and we adopt that relation as the organizing principle of the revised manuscript. The pipeline described in this section is *exploratory*: its design was fixed while observing the evaluation protocol of Section 4, and every number it produces is a development result. The evaluation of Section 4.10.3 is *confirmatory*: the architecture and the feature map were fixed in advance to the specification of Table 1; training, validation, and test data are drawn from three independent seed streams that are never mixed; and the test stream was evaluated once and used for nothing else. The confirmatory evaluation reproduces the headline reduction (95.1% against the exploratory pipeline’s 95.4%), the concentration of the advantage in n<k, and the k=2 instability. Where the two disagree, the confirmatory figures are the ones on which the reader should rely. What the confirmatory evaluation does not do is retroactively lock the exploratory pipeline, and we make no such claim: the exploratory results are reported because they carry the multi-estimator comparison and the ablations, not because they are protected from experimenter-level overfitting.

**Remark** **1.** *Two caveats delimit what the offline strategy does and does not guarantee. First, the weights λ(φ;θ) are functions of the FoF vector, which also determines every base estimate (Lemma 1); weights and estimates are therefore statistically dependent, and no oracle or regret guarantee follows from the offline construction alone; deriving one is the main theoretical problem left open by this work. Second, the loss (5) is minimized under the uniform null, so the fitted mapping is calibrated for the testing problem “$H=H_{\max}$ versus nearby alternatives” rather than for general-purpose entropy estimation. Nothing in the construction prevents a sample-dependent combination from returning $H_{\max}$ on every sample; Section 4.5 supplies the behavioral evidence that the fitted model does not.*

### 3.4. Theoretical Analysis

Although the offline construction yields no guarantee by itself (Remark 1), the mixture admits an oracle inequality that identifies exactly what the weight mapper must learn and how errors in learning it propagate. Write $e_{j}={\hat{H}}_{j}-H$ for the error of base estimator *j*, and let

(6) $$M\left(\varphi \right)\;=\;E\left[\;e\;e^{⊤}|\varphi \;\right]\in R^{M\times M}$$

be the conditional second-moment matrix of the base errors given the occupancy-profile features. For any measurable weight function $\lambda :R^{37}\to \Delta^{M}$, the risk of the induced mixture is $R\left(\lambda \right)=E\left[\lambda {\left(\varphi \right)}^{⊤}M\left(\varphi \right)\lambda \left(\varphi \right)\right]$, and we call $\lambda^{*}\left(\varphi \right)=\arg\min_{\lambda \in \Delta^{M}}\lambda^{⊤}M\left(\varphi \right)\lambda$ the *sample-conditional oracle*, with risk $R^{*}$.

**Theorem** **1** (Oracle inequality for conditional mixtures)**.** *Write ${∥M∥}_{\max}=\max_{i,j}\left|M_{ij}\right|$, let $R^{*}\left(\varphi \right)=\lambda^{*⊤}M\left(\varphi \right)\lambda^{*}$, and define the* suboptimality gaps *$g_{j}\left(\varphi \right)={\left(M\left(\varphi \right)\lambda^{*}\left(\varphi \right)\right)}_{j}-R^{*}\left(\varphi \right)$, which are non-negative and vanish on the support of $\lambda^{*}$. Then, for every measurable $\lambda :R^{37}\to \Delta^{M}$,*

(7) $$0\;\leq \;R\left(\lambda \right)-R^{*}\;\leq \;E\left[{\;∥M\left(\varphi \right)∥}_{\max}\;{∥\lambda \left(\varphi \right)-\lambda^{*}\left(\varphi \right)∥}_{1}^{2}\;+\;2\;\langle g(\varphi ),\;\lambda (\varphi )\rangle \right].$$

*In particular, if $\lambda^{*}\left(\varphi \right)$ has full support for almost every **φ**, then g≡0, and the excess risk is* quadratic *in the approximation error: $R\left(\lambda \right)-R^{*}\leq {E[∥M\left(\varphi \right)∥}_{\max}∥\lambda -\lambda^{*}{∥}_{1}^{2}]$.*

**Proof.**  Fix φ, and set $\delta =\lambda -\lambda^{*}$, so that $\lambda^{⊤}M\lambda -\lambda^{*⊤}M\lambda^{*}=\delta^{⊤}M\delta +2{\left(M\lambda^{*}\right)}^{⊤}\delta$. For the first term, Hölder gives $\delta^{⊤}M\delta \leq {∥\delta ∥}_{1}{∥M\delta ∥}_{\infty }\leq {∥M∥}_{\max}{∥\delta ∥}_{1}^{2}$. For the second, $1^{⊤}\delta =0$; hence, ${\left(M\lambda^{*}\right)}^{⊤}\delta =\sum_{j}\left[{\left(M\lambda^{*}\right)}_{j}-R^{*}\right]\delta_{j}=\langle g,\delta \rangle =\langle g,\lambda \rangle$, the last equality because $g_{j}=0$ whenever $\lambda_{j}^{*}>0$, by the Karush–Kuhn–Tucker conditions for minimizing the convex quadratic $\lambda \mapsto \lambda^{⊤}M\lambda$ over $\Delta^{M}$; the same conditions give $g_{j}\geq 0$ for all *j*, which also yields the left-hand inequality. Taking expectations completes the proof.    □

Theorem 1 converts the estimation problem into a regression problem: the excess risk of ASED over the sample-conditional oracle is controlled by the error with which the network approximates $\lambda^{*}$, scaled by the local geometry of M(φ). Two features matter for what follows. The multiplier is ${∥M∥}_{\max}$ rather than the cruder $\max_{j}E\left[e_{j}^{2}|\varphi \right]$, which it never exceeds; and when the conditional oracle has full support, the dependence on the approximation error is quadratic, so moderate weight errors are second-order. Whether that full-support condition holds in the small-sample regimes studied here is not established empirically: the softmax output assigns strictly positive weights by construction, which says nothing about the geometry of $\lambda^{*}\left(\varphi \right)$, and we do not estimate $\lambda^{*}$ directly. The bound also explains why the loss (5) must be balanced across regimes: ${∥M\left(\varphi \right)∥}_{\max}$ varies by orders of magnitude between n≪k and n≫k, so an unweighted objective concentrates all effort where the multiplier is largest. One limit of Theorem 1 should be stated rather than left to inference, since it is easy to over-read. The inequality bounds the excess risk of a mixture in terms of the $L_{1}$ error of *its own* weights; it does not assert that the offline fit of this section makes that error small, and no bound on $E∥\lambda \left(\varphi ;\theta \right)-\lambda^{*}{\left(\varphi \right)∥}_{1}$ for the trained mapper is established anywhere in this paper. The theorem therefore identifies what would have to be shown for a performance guarantee to follow, and the empirical results of Section 4 are evidence about the unproved quantity rather than a consequence of the proved one. No theoretical result in this section should be read as a guarantee for the deployed estimator.

The next result gives a checkable condition under which weights conditioned on the regime alone are useless; Section 4 verifies empirically at which sample sizes the condition actually holds and reports one size at which it fails.

**Corollary** **1** (A covariance condition excluding fixed-mixture improvement)**.** *Let $j^{*}\in \arg\min_{j}M_{jj}$, and suppose*

(8) $$M_{ij^{*}}\;\geq \;M_{j^{*}j^{*}}\;\mathrm{for}\;\mathrm{every}\;i=1,\ldots ,M,$$

*equivalently, whenever $M_{ii}M_{j^{*}j^{*}}>0$ so that the correlation is defined, $\rho_{ij^{*}}\geq \sqrt{M_{j^{*}j^{*}}/M_{ii}}$. Then, $\min_{\lambda \in \Delta^{M}}\lambda^{⊤}M\lambda =M_{j^{*}j^{*}}$: no convex combination with fixed weights improves on the best single estimator. For M=2, condition (8) reduces to $\rho \geq \sqrt{M_{11}/M_{22}}$ with $M_{11}\leq M_{22}$.*

**Proof.**  The objective $f\left(\lambda \right)=\lambda^{⊤}M\lambda$ is convex on $\Delta^{M}$ because *M* is positive semidefinite, so the vertex $e_{j^{*}}$ is a global minimizer if and only if the first-order condition $\nabla f{\left(e_{j^{*}}\right)}^{⊤}\left(\lambda -e_{j^{*}}\right)\geq 0$ holds for every $\lambda \in \Delta^{M}$. Since $\nabla f\left(e_{j^{*}}\right)=2Me_{j^{*}}$, this is equivalent to ${\left(Me_{j^{*}}\right)}_{i}\geq {\left(Me_{j^{*}}\right)}_{j^{*}}$ for all *i*, i.e., to (8). Writing $M_{ij^{*}}=\rho_{ij^{*}}\sqrt{M_{ii}M_{j^{*}j^{*}}}$ gives the correlation form.    □

Condition (8) has a transparent reading: the column of *M* belonging to the best estimator dominates its own diagonal entry, so every other estimator has a sufficiently large positive error cross-moment with the best one, and adding it to the mixture cannot cancel error on average. Its scope is correspondingly narrow, and we state the boundary explicitly. Corollary 1 concerns *fixed* mixtures within a regime: where the condition holds, no regime-indexed convex combination of the base set improves on its best member, and nothing further follows. It does not establish that sample-conditional weighting is superior, it does not show that the sample-conditional oracle strictly dominates the per-regime one, and it says nothing about the fitted mapper. Its role is to remove one competing explanation for the observed gain, that a fixed mixture would have sufficed, and not to supply a positive argument for the method.

Condition (8) concerns fixed mixtures within a regime, so the relevant matrix is the *regime-conditional* second-moment matrix

(9) $${\bar{M}}_{k,n}\;:=\;E\left[ee^{⊤}\;|\;K=k,\;N=n\right],$$

obtained from M(φ) by averaging over samples of a given size, and it is ${\bar{M}}_{k,n}$ not M(φ) pointwise to which the corollary is applied here. The condition involves every base estimator, so we verify it directly. Table 2 reports the ratios $M_{ij^{*}}/M_{j^{*}j^{*}}$ estimated from 4000 replications at each critical sample size for k=256.

Since the entry $i=j^{*}$ equals one by construction, the binding quantity is the smallest off-diagonal ratio $\min_{i\neq j^{*}}{\bar{M}}_{ij^{*}}/{\bar{M}}_{j^{*}j^{*}}$, which we report with a percentile bootstrap over the 4000 replications (B=4000 resamples of the replication index). At n=8, it is 3.77 (SHR) with a lower bound of 3.40, and at n=64, it is 1.84 (LAP) with a lower bound of 1.72: the condition is satisfied and certified at the 95% level. At n=16, the plug-in estimate satisfies the condition at 1.07 (SHR), but its bootstrap lower bound is 0.989, so the empirical certification is inconclusive. At n=32, the condition *fails*: the smallest off-diagonal ratio is 0.48 (LAP), with lower bound 0.44. Solving $\min_{\lambda \in \Delta^{M}}\lambda^{⊤}M\lambda$ numerically there confirms and quantifies the failure: the optimal fixed mixture puts weight (0.66,0.34) on (SHR, LAP) and attains $1.85\times 10^{-3}$ against $2.22\times 10^{-3}$ for the best single estimator, a 17% improvement. ASED attains $6.5\times 10^{-4}$ at the same size, so sample-conditional weighting still helps substantially, but at n=32, it improves on an achievable fixed mixture rather than on an unbeatable baseline. We state this explicitly because n=32 is one of the sizes where ASED’s advantage is largest, and the necessity argument does not cover it.

The ablation of Section 4.7 confirms both halves of this prediction empirically.

The results so far concern the best achievable mixture. For deployment, one also wants guarantees that do not depend on the distribution used to train the weights. Theorem 2 establishes consistency for fixed-alphabet i.i.d. full-support sources; Propositions 1 and 2 then give non-asymptotic safety bounds, the first of which transmits consistency only when the base estimators are themselves consistent under the source considered.

**Theorem** **2** (Consistency and when asymptotic equivalence holds)**.** *Fix k, and let $X_{1},X_{2},\ldots$ be i.i.d. from P with $p_{x}>0$ for all x∈K. Then, for* every *weight function $\lambda :R^{37}\to \Delta^{M}$,*

*(i)*  *(Consistency) ${\hat{H}}_{\mathrm{ASED}}\overset{P}{\to }H$;*
*(ii)*  *(Expansion) writing $\lambda_{\mathrm{MIN}}$ for the weight on the minimax-prior estimator,*
(10) $${\hat{H}}_{\mathrm{ASED}}-{\hat{H}}_{\mathrm{ML}}\;=\;\lambda_{\mathrm{MIN}}\left(\varphi_{n}\right)\;O_{P}\left(n^{-1/2}\right)\;+\;O_{P}\left(n^{-1}\right);$$
*(iii)*  *(Asymptotic normality) if in addition $\sigma^{2}=\mathrm{Var}\left(\log_{2}p_{X}\right)>0$ and $\lambda_{\mathrm{MIN}}\left(\varphi_{n}\right)\overset{P}{\to }0$, then $\sqrt{n}\left({\hat{H}}_{\mathrm{ASED}}-H\right)\overset{d}{\to }N\left(0,\sigma^{2}\right)$, matching the plug-in estimator to first order.*

**Proof.**  Each Dirichlet member satisfies ${\hat{p}}^{\left(a_{n}\right)}=\left(1-\alpha_{n}\right)\hat{p}+\alpha_{n}u$ with $\alpha_{n}=ka_{n}/\left(n+ka_{n}\right)$ and u uniform. For the bounded-pseudocount Dirichlet members of the ASED base set, LAP and SG, the pseudocount $a_{n}$ is bounded, so $\alpha_{n}=O\left(n^{-1}\right)$ (the same observation applies to the JEF comparison estimator, which is not a member of the mixture); since $t\mapsto -t\log_{2}t$ is Lipschitz on compact subsets of (0,1] and $\min_{x}{\hat{p}}_{x}\to \min_{x}p_{x}>0$ almost surely, these estimators differ from ${\hat{H}}_{\mathrm{ML}}$ by $O_{P}\left(n^{-1}\right)$. The same holds for MM, whose correction is exactly $\left(m_{\mathrm{obs}}-1\right)/\left(2n\ln2\right)$, and for CS, because $f_{1}=0$ eventually almost surely. For SHR, two cases must be separated. If P≠U, then $\sum_{x}{\left(1/k-{\hat{p}}_{x}\right)}^{2}\to \sum_{x}{\left(1/k-p_{x}\right)}^{2}>0$, so the denominator of ${\hat{\lambda }}^{*}$ grows linearly while the numerator stays bounded, giving ${\hat{\lambda }}^{*}=O_{P}\left(n^{-1}\right)$ and, hence, ${\hat{H}}_{\mathrm{SHR}}-{\hat{H}}_{\mathrm{ML}}=O_{P}\left(n^{-1}\right)$. If P=U, then $\sum_{x}{\left(1/k-{\hat{p}}_{x}\right)}^{2}=O_{P}\left(n^{-1}\right)$, and ${\hat{\lambda }}^{*}$ need not vanish, but the conclusion holds for a different reason. Writing $q_{n}=\left(1-{\hat{\lambda }}^{*}\right){\hat{p}}_{n}+{\hat{\lambda }}^{*}u$ and expanding *both* entropies around u where the gradient of *H* is constant on the simplex, so the linear terms cancel gives

$$H\left(q_{n}\right)-H\left({\hat{p}}_{n}\right)=\frac{1}{2}{\left(q_{n}-u\right)}^{⊤}\nabla^{2}H\left(u\right)\left(q_{n}-u\right)-\frac{1}{2}{\left({\hat{p}}_{n}-u\right)}^{⊤}\nabla^{2}H\left(u\right)\left({\hat{p}}_{n}-u\right)+o_{P}\left(n^{-1}\right),$$

and since $∥q_{n}-u∥\leq ∥{\hat{p}}_{n}-u∥=O_{P}\left(n^{-1/2}\right)$, both quadratic forms are $O_{P}\left(n^{-1}\right)$. For MIN, however, $a_{n}=\frac{\sqrt{n}}{k}$ gives $\alpha_{n}=\sqrt{n}/\left(n+\sqrt{n}\right)≍n^{-1/2}$, and a first-order Taylor expansion of *H* at $\hat{p}$ yields ${\hat{H}}_{\mathrm{MIN}}-{\hat{H}}_{\mathrm{ML}}=-\alpha_{n}\sum_{x}\left(1/k-{\hat{p}}_{x}\right)\left(\log_{2}{\hat{p}}_{x}+\log_{2}e\right)+O_{P}\left(\alpha_{n}^{2}\right)$, the sign following from $\partial H/\partial p_{x}=-\left(\log_{2}p_{x}+\log_{2}e\right)$ together with ${\hat{p}}^{\;\mathrm{MIN}}=\hat{p}+\alpha_{n}\left(u-\hat{p}\right)$, whose leading term does not vanish unless *P* is uniform. Hence, ${\hat{H}}_{\mathrm{MIN}}-{\hat{H}}_{\mathrm{ML}}=O_{P}\left(n^{-1/2}\right)$ in general, and $\Theta_{P}\left(n^{-1/2}\right)$ whenever the limiting linear term $\sum_{x}\left(1/k-p_{x}\right)\left(\log_{2}p_{x}+\log_{2}e\right)$ is non-zero, which gives (ii) because $\lambda \in \Delta^{M}$ bounds the contribution of every other member by $O_{P}\left(n^{-1}\right)$. Statement (i) follows since every base estimator is consistent for fixed *k* and a convex combination of consistent estimators is consistent. Statement (iii) follows from (ii), the delta method applied to ${\hat{H}}_{\mathrm{ML}}$, and Slutsky’s lemma.    □

**Remark** **2** (Sharpness)**.** *Part (ii) cannot be improved to $O_{P}\left(n^{-1}\right)$ without a condition on $\lambda_{\mathrm{MIN}}$: taking $\lambda_{\mathrm{MIN}}\equiv 1$ makes ASED equal to MIN. Simulation confirms the rate: for a fixed non-uniform P on k=8, the mean of $|{\hat{H}}_{\mathrm{MIN}}-{\hat{H}}_{\mathrm{ML}}|$ multiplied by $\sqrt{n}$ is nearly constant (1.65 to 1.78 for $n=10^{2}$ to $10^{5}$), whereas the same quantity for LAP and SHR multiplied by n is constant (14.3 and 9.9). Two caveats govern the applicability of part (iii). First, it requires $\sigma^{2}=\mathrm{Var}\left(\log_{2}p_{X}\right)>0$, which* fails under the uniform null, *where $p_{X}\equiv 1/k$ and $\sigma^{2}=0$; part (iii) therefore does not apply to the byte experiments of Section 4, whose source is uniform by design, and the degenerate case would require a second-order analysis that we do not carry out. Second, on the tested byte grid, the fitted MIN weights are empirically negligible, which is consistent with, but does not verify, the asymptotic vanishing-weight condition: small weights on a finite grid of n do not establish $\lambda_{\mathrm{MIN}}\left(\varphi_{n}\right)\overset{P}{\to }0$. For bits, MIN receives substantial weight and the condition plainly does not hold.*

Part (i) is the consistency statement the construction needs: no assumption on how the weights were obtained enters the argument, only that they live in the simplex. We do not offer it as a substantive result. Every base estimator is consistent at fixed *k*, and a convex combination of consistent estimators is consistent by an elementary argument; part (i) is therefore a sanity property, confirming that the mixture cannot destroy something its ingredients already have, rather than evidence in favor of sample-conditional weighting. It is recorded because the weights here are learned and could in principle be arbitrary, so ruling out pathological behavior of an arbitrary simplex-valued rule is worth doing once, cheaply. Parts (ii)–(iii) delimit the method from the other side. Under non-uniform *P*, positive asymptotic variance, and a vanishing MIN weight, ASED has the same first-order asymptotic distribution as the plug-in estimator; thus, in that regime, its value lies entirely in the short-sample behavior, where the correction terms dominate, precisely the regime this paper studies. We make no claim about second-order or constant-factor behavior and none about the degenerate uniform case where the first-order expansion vanishes. The following non-asymptotic counterpart is elementary two lines of Jensen, but it is what rules out arbitrary behavior at finite *n* on sources far from the training distribution. It bounds the mixture from above and is not a guarantee relative to the worst base estimator.

**Proposition** **1** (Distribution-free safety upper bound)**.** *Let P be any source distribution on K with entropy H. For every weight function $\lambda :R^{37}\to \Delta^{M}$,*

(11) $${\mathrm{MSE}}_{P}\left({\hat{H}}_{\mathrm{ASED}}\right)\;\leq \;E\left[\max_{1\leq j\leq M}e_{j}^{2}\right]\;\leq \;\sum_{j=1}^{M}{\mathrm{MSE}}_{P}\left({\hat{H}}_{j}\right)\;\leq \;M\max_{1\leq j\leq M}{\mathrm{MSE}}_{P}\left({\hat{H}}_{j}\right).$$

*Consequently, (i) if every base estimator is consistent for H under P, so is ASED; (ii) if ${\mathrm{MSE}}_{P}\left({\hat{H}}_{j}\right)=O\left(r_{n}\right)$ for every j, then ${\mathrm{MSE}}_{P}\left({\hat{H}}_{\mathrm{ASED}}\right)=O\left(r_{n}\right)$; and (iii) both conclusions hold irrespective of the distribution on which the weights were trained.*

**Proof.**  Conditionally on the sample, ${\hat{H}}_{\mathrm{ASED}}-H=\sum_{j}\lambda_{j}e_{j}$ with $\lambda \in \Delta^{M}$. Convexity of $t\mapsto t^{2}$ (Jensen) gives ${\left(\sum_{j}\lambda_{j}e_{j}\right)}^{2}\leq \sum_{j}\lambda_{j}e_{j}^{2}\leq \max_{j}e_{j}^{2}\leq \sum_{j}e_{j}^{2}$ pointwise, and taking expectations under *P* yields the three displayed inequalities. Statements (i)–(iii) follow because no property of λ beyond membership in the simplex is used.    □

**Remark** **3** (Why the maximum sits inside the expectation)**.** *For weights fixed in advance, the first bound would read $\sum_{j}\lambda_{j}{\mathrm{MSE}}_{P}\left({\hat{H}}_{j}\right)\leq \max_{j}{\mathrm{MSE}}_{P}\left({\hat{H}}_{j}\right)$, without the factor M. That simplification is unavailable here: λ=λ(φ) is a function of the same sample as the base estimates, so $E[\sum_{j}\lambda_{j}\left(\varphi \right)e_{j}^{2}]$ cannot be split into $\sum_{j}\lambda_{j}E\left[e_{j}^{2}\right]$, and the maximum must remain inside the expectation, where $E\left[\max_{j}e_{j}^{2}\right]\geq \max_{j}E\left[e_{j}^{2}\right]$. The factor M is thus the price of data-dependent weighting; it is benign for the asymptotic conclusions (i)–(ii), which is all we claim.*

Proposition 1 is a worst-case guarantee. Conditioning on φ before taking expectations sharpens it into a bound relative to the *best* base estimator, at the price of an explicit dependence on how much weight the mapper misplaces.

**Proposition** **2** (Bound relative to the conditionally best estimator)**.** *For each **φ**, let $j^{*}\left(\varphi \right)\in \arg\min_{j}E\left[e_{j}^{2}|\varphi \right]$, and let $\varepsilon \left(\varphi \right)=1-\lambda_{j^{*}\left(\varphi \right)}\left(\varphi \right)$ be the weight the mixture places away from it. Then,*

(12) $${\mathrm{MSE}}_{P}\left({\hat{H}}_{\mathrm{ASED}}\right)\;\leq \;\underset{R_{\mathrm{sel}}}{\underset{︸}{E\left[\min_{j}E\left[e_{j}^{2}|\varphi \right]\right]}}\;+\;E\left[\varepsilon \left(\varphi \right)\;\max_{j}E\left[e_{j}^{2}|\varphi \right]\right],$$

*where $R_{\mathrm{sel}}\leq \min_{j}{\mathrm{MSE}}_{P}\left({\hat{H}}_{j}\right)$ is the risk of the conditionally optimal* selection *rule.*

**Proof.**  Conditionally on φ the weights are constants, so Jensen and then linearity of the conditional expectation give

$$E\left[{\left(\sum_{j}\lambda_{j}e_{j}\right)}^{2}\;|\;\varphi \right]\;\leq \;\sum_{j}\lambda_{j}\left(\varphi \right)\;E\left[e_{j}^{2}|\varphi \right]\;\leq \;\min_{j}E\left[e_{j}^{2}|\varphi \right]+\varepsilon \left(\varphi \right)\max_{j}E\left[e_{j}^{2}|\varphi \right],$$

the second step splitting the sum at $j^{*}\left(\varphi \right)$ and bounding the remaining weights, which total ε(φ). Taking expectations over φ gives the display. Conditioning first is essential: the pointwise bound $\sum_{j}\lambda_{j}e_{j}^{2}\leq \max_{j}e_{j}^{2}$ would lead to $E[\max_{j}e_{j}^{2}]$, which exceeds $\max_{j}E\left[e_{j}^{2}\right]$. Finally, $R_{\mathrm{sel}}\leq \min_{j}{\mathrm{MSE}}_{P}\left({\hat{H}}_{j}\right)$, because selecting the conditionally best estimator is at least as good as committing to any fixed one.    □

Proposition 2 describes two limiting behaviors. If the fitted mapper places asymptotically all its weight on the conditionally best estimator (ε→0), its risk approaches $R_{\mathrm{sel}}$; when it spreads weight, the excess is controlled by ε times the worst conditional risk, and Proposition 1 is recovered as the crude case ε≤1. We do not estimate $R_{\mathrm{sel}}$ in the present experiments, and two empirical readings must therefore be avoided. The concentration on SHR observed for n≥64 shows convergence towards SHR but does not establish that SHR is the conditionally best estimator for almost every feature vector, which would require estimating the conditional risks $r_{j}\left(\varphi \right)=E\left[e_{j}^{2}|\varphi \right]$. Likewise, ASED’s improvement over the best fixed estimator at n≤32 does not by itself imply improvement over $R_{\mathrm{sel}}$, since $R_{\mathrm{sel}}\leq \min_{j}{\mathrm{MSE}}_{P}\left({\hat{H}}_{j}\right)$ makes the conditional selector the stronger benchmark.

Proposition 1 is what makes offline calibration safe to deploy, provided it is read correctly. For *every individual sample*, the squared error of the convex mixture cannot exceed the largest squared error among its base estimators. In expectation, this yields the displayed bound and nothing stronger: since $E\left[\max_{j}e_{j}^{2}\right]\geq \max_{j}E\left[e_{j}^{2}\right]$, the proposition does *not* guarantee that the MSE of ASED is below the MSE of the worst base estimator. It also predicts the shape of the failure documented in Section 4.9 on a strongly skewed source. ASED degrades *to* the level of the estimators it leans on, not below it, and this is what we observe. Estimating the first bound directly over 2000 replications at n=256: at Zipf s=1.0, the measured $\mathrm{MSE}({\hat{H}}_{\mathrm{ASED}})=1.170$ against $E[\max_{j}e_{j}^{2}]=1.184$, so the bound is nearly saturated, whereas under the uniform null, the same comparison reads $2\times 10^{-5}$ against 0.659: loose by four orders of magnitude. The bound is therefore informative exactly where the method is weakest and uninformative where it is strongest, which is the expected behavior of a worst-case guarantee.

Finally, an idealized version of the offline fit admits a standard uniform-convergence guarantee. Because it bounds an empirical-risk minimizer related to, but distinct from, the procedure actually run, and because the resulting numerical bound is vacuous at our training size, we state it and evaluate its complexity term in Appendix B, recording here only the consequence.

**Remark** **4** (Approximation, generalization, optimization, and design mismatch)**.** *Combining Theorem 1 with Proposition A1 of Appendix B,*

(13) $$\begin{array}{cc} R\left(\hat{\lambda }\right)-R^{*}\; & \;\leq \;\underset{\mathrm{approximation}}{\underset{︸}{2\;E\;\left[∥\lambda^{\Lambda }-\lambda^{*}{∥}_{1}\max_{j}E\left[e_{j}^{2}|\varphi \right]\right]}}\;+\;\underset{\mathrm{generalization}\;\left(\mathrm{idealized}\right)}{\underset{︸}{8B\;R_{N}\left(F_{\Lambda }\right)+2B^{2}\sqrt{2\log(2/\delta )/N}}} \end{array}$$

(14) $$\begin{array}{cc} \; & \;+\underset{\mathrm{optimization}}{\underset{︸}{\varepsilon_{\mathrm{opt}}}}+\underset{\mathrm{stratification}}{\underset{︸}{\varepsilon_{\mathrm{design}}}}, \end{array}$$

*where $\lambda^{\Lambda }$ attains $\inf_{\Lambda }R$. The first term is an approximation-theoretic question about how well a small network represents the conditional oracle on the FoF domain; the second is controlled by the training budget. Only the first two terms are analyzed here. The generalization term is the idealized PAC quantity of Appendix B, which bounds an empirical-risk minimizer related to, but distinct from, the deployed training procedure; the optimization error $\varepsilon_{\mathrm{opt}}$ of Adam on a non-convex objective and the design-mismatch term $\varepsilon_{\mathrm{design}}$ from stratified sampling with a regime-weighted loss are both uncontrolled. Appendix B evaluates the complexity term numerically, by a norm-based argument over a data-dependent class and by Monte Carlo; neither is a valid uniform bound for the deployed model.*

### 3.5. Adaptive Inference

Algorithm 1 formalizes inference. Total complexity is O(n+k), dominated by the count computation and matching the complexity of any plug-in estimator.
**Algorithm 1** ASED Inference**Require:** Sample $x=(x_{1},\ldots ,x_{n})$, alphabet size *k*, trained weights θ**Ensure:** Entropy estimate ${\hat{H}}_{\mathrm{ASED}}$  1: Compute $y_{x}=\left|\left\{i:x_{i}=x\right\}\right|$ for all x∈K
  2: Compute FoF sequence $\left\{f_{i}\right\}$ and feature vector φ
*// O(n+k)*  3: Evaluate base estimators: ${\hat{H}}_{j}$ for j∈B
*// O(n+k) each*  4: $\lambda \leftarrow \phi_{\theta }\left(\varphi \right)$
*// O(1) — fixed network*  5: ${\hat{H}}_{\mathrm{ASED}}\leftarrow \sum_{j}\lambda_{j}{\hat{H}}_{j}$
  6: **return** ${\hat{H}}_{\mathrm{ASED}}$


## 4. Experimental Evaluation

### 4.1. Protocol

The null-protocol design follows Contreras Rodríguez et al. [2]. For each combination of alphabet size k∈{2,256} and sample size n∈{8,16,32,64,128,256,512,1024,2048,4096,8192,16384}, we generate 1000 independent samples drawn uniformly at random with a fixed-seed NumPy generator, disjoint from the training draws. We re-implement eight baseline estimators spanning the plug-in, corrected, Bayesian, coverage-based, and shrinkage families of that comparison (ML, MM, LAP, JEF, SG, MIN, CS, SHR) and the general-purpose NSB estimator [18], computed by numerical integration of the Wolpert–Wolf posterior mean entropy against the NSB prior over a 60-point grid, together with ASED and the constant control ${\hat{H}}_{\mathrm{CONST}}\equiv H_{\max}$. Performance is measured by

(15) $$\mathrm{Bias}\left(\hat{H}\right)=H_{\max}-E\left[\hat{H}\right],\;\mathrm{MSE}\left(\hat{H}\right)=\mathrm{Bias}{\left(\hat{H}\right)}^{2}+\mathrm{Var}\left(\hat{H}\right),$$

and the *integrated MSE* $\sum_{n}\mathrm{MSE}\left(n\right)$ summarizes performance across the 12 sample sizes. Uncertainty is quantified by percentile bootstrap intervals over the 1000 replications (B=2000); the bootstrap is used for intervals only, and the ASED–SHR integrated-MSE comparison is supplemented by a paired randomization test under the sharp null of pairwise exchangeability. Tables report representative columns. One rule of run hygiene, prompted by the inconsistency documented in Section 4.11, is now enforced throughout and stated here so that every table below can be read against it: each table and each aggregate reported in this paper is computed from a single evaluation run, and figures from different runs are never combined within a single statement. Where a quantity was previously taken from a run other than the one behind its own table, it has been withdrawn and replaced either by the value implied by that table itself or by a value from a single internally consistent run, identified as such at the point of use. Regime splits use the disjoint partition n<k, k≤n≤512, n>512 throughout; the overlapping definitions used in the previous version are retired.

### 4.2. Results: Byte Sequences (K=256)

Table 3 reports |Bias| for representative estimators. ASED achieves a bias of 0.002 bits at n=8, the lowest of all estimators at the smallest sample size, versus 0.050 for SHR. At n=8 the mean weights are approximately 0.90 on SHR and 0.10 on LAP, moving to (0.60,0.40) at n=16 and back toward SHR from n=32 onward (Figure 1); for n≥64, ASED and SHR coincide, confirming that SHR is essentially optimal within the evaluated comparison set in the large-sample regime and that the mixture does not degrade it.

Table 4 reports MSE in scientific notation, including the constant control. Full-precision per-size results underlying this and every subsequent table through the cryptographic-source results of Section 4.10 are released as Supplementary Materials (Data S2). The integrated MSE for ASED is $1.7\times 10^{-3}$ with bootstrap 95% CI $\left[1.6,1.9\right]\times 10^{-3}$, against $3.83\times 10^{-2}$ for SHR with CI $\left[3.1,4.6\right]\times 10^{-2}$: a 95.4% reduction (21.9×; percentages and ratios are computed at full precision: $1.7462\times 10^{-3}$ versus $3.8256\times 10^{-2}$, a reduction of 95.4354%, and only the presented result is rounded), with disjoint intervals. A paired difference is the more direct summary. The value consistent with Table 4 is $\Delta_{\mathrm{MSE}}={\mathrm{MSE}}_{\mathrm{ASED}}-{\mathrm{MSE}}_{\mathrm{SHR}}=-3.651\times 10^{-2}$, obtained from the full-precision integrated values of that table and from no other source. The figure previously reported here, $-3.69\times 10^{-2}$ with interval $\left[-4.81\times 10^{-2},\;-2.73\times 10^{-2}\right]$, was computed on a different evaluation run; it has been withdrawn rather than carried forward with a caveat, and its cause is documented in Section 4.11. The paired bootstrap interval this paper reports is therefore the one produced by the single internally consistent run detailed in Section 4.11, $-3.416\times 10^{-2}$ with a 95% interval $\left[-4.335\times 10^{-2},\;-2.666\times 10^{-2}\right]$, which excludes zero, and it is quoted there together with the run that produced it rather than alongside the values tabulated here. Because ordinary bootstrap resamples are generated around the observed empirical distribution rather than under the null, the frequency with which they reverse the ordering is a stability diagnostic and not a calibrated *p*-value; we therefore report a paired randomization test instead. ASED and SHR are evaluated on the same samples, so the two labels may be swapped within each paired replication. Under the sharp null of pairwise exchangeability of the estimator errors, the paired randomization test yielded no assignment as extreme as the observed statistic over 20,000 independent sign-flip assignments (0/20,000; $\hat{p}=5.0\times 10^{-5}$ with the plus-one correction), the largest randomized difference being $1.7\times 10^{-2}$ against an observed $3.7\times 10^{-2}$. Exchangeability is a stronger hypothesis than equality of integrated MSE; two estimators can share an MSE without exchangeable error distributions so the *p*-value refers to that sharp null; the disjoint bootstrap intervals reported above are the evidence bearing directly on the MSE comparison. The next best baseline, LAP ($1.08\times 10^{-1}$), is 62× worse than ASED; NSB, evaluated under the same full protocol, reaches $1.12\times 10^{1}$, dominated by the deep undersample regime (7.7 at n=8) where its general-purpose prior cannot exploit the uniform target. The CONST row makes the protocol’s blind spot explicit: the constant rule achieves zero error by construction under the null, which is precisely why Section 4.5 and Section 4.6 are part of the evaluation.

Figure 2 shows mean estimates across sample sizes; ASED tracks $H_{\max}=8$ closely even at the smallest *n*, while the baselines diverge for n<k. Figure 3 reports the gap against the per-regime *best-single-estimator benchmark* (the best single estimator chosen with knowledge of the regime; not to be confused with the sample-conditional oracle $\lambda^{*}\left(\varphi \right)$): the gap is negative for n≤32, as ASED is more accurate than any evaluated single estimator chosen with knowledge of the regime, and zero thereafter. The mechanism is sample-conditional weighting rather than estimator selection: at n=8, the errors of SHR and LAP are strongly positively correlated (ρ≈0.995), so no fixed convex combination of the base set could beat the better endpoint; the gain comes from weights that covary with the FoF features predicting the plug-in error, an implicit learned bias correction under the null. This behavior is consistent with null calibration and is what makes the power analysis below indispensable rather than optional. Figure 4 summarizes the two preceding comparisons in a single head-to-head panel, plotting integrated MSE for all evaluated estimators on a log scale across byte sequences: ASED (red) tracks the lower envelope of the base set throughout the sample-size range.

### 4.3. Results: Bit Sequences (K=2)

Table 5 reports the complete ranking for bits. ASED attains integrated MSE 0.0135 with bootstrap interval [0.0122,0.0147], a 31% improvement over SHR (0.0196) and better than LAP (0.0158), ranking second behind the minimax-prior estimator MIN (0.0109), whose interval does not overlap ASED’s. Bit-sequence numbers quoted elsewhere in this paper come from auxiliary runs with fewer replications or from separately retrained mappers, reported in the alphabet-generalization and extended-comparison tables introduced in Section 4.6 and Section 4.8; the spread across those runs, roughly 0.007 to 0.022, reflects both Monte Carlo error at 200–400 replications and genuine retraining variability at k=2, where the FoF descriptor is nearly degenerate. This instability is itself a limitation of the method for tiny alphabets, and Table 5 is the locked reference. The residual gap to MIN is a genuine limitation: MIN belongs to ASED’s base set, so an additional improvement was available within the mixture’s hypothesis class that the fitted mapper only partially realized. The plausible cause is feature degeneracy: for k=2, the FoF histogram has at most two non-zero coordinates, so the occupancy features carry little regime information beyond the count imbalance. Augmenting the descriptor with low-*k* statistics is a natural remedy, which we leave to future work. We therefore make no performance claim for bit sequences, and the applicability statements of Section 5 are confined to bytes. Three facts jointly support that restriction. ASED does not outperform MIN, an estimator in its own base set, so the mixture fails to achieve an improvement available within its hypothesis class. The run-to-run spread across the auxiliary protocols, roughly 0.007 to 0.022 in integrated MSE, is of the same order as the margins separating the estimators in Table 5, so the ranking below the top position is not stable. Furthermore, the 31% improvement over SHR quoted elsewhere is a single-run figure at one alphabet, not a property of the method. At k=2, the honest summary is that ASED is competitive with, but not better than, the best classical estimator available.

### 4.4. Sensitivity of the Headline Number to the Aggregation Scheme

Integrated MSE weights the twelve sample sizes equally on a linear scale, which lets the smallest *n* dominate. Since the headline reduction depends on that choice, we report it under four schemes (bytes, ASED versus SHR): sum over the grid, 95.4%; arithmetic mean, 95.4%; trapezoidal integration uniform in $\log_{2}n$, 92.9%; and the least favorable, the mean over *n* of the *per-size ratio* ${\mathrm{MSE}}_{\mathrm{ASED}}\left(n\right)/{\mathrm{MSE}}_{\mathrm{SHR}}\left(n\right)$, which is 0.76, i.e., a reduction of only 24%. Split by regime, the picture is unambiguous, and it is now reported from a single internally consistent run against a disjoint partition, replacing the mixed-run figures that appeared here in the previous version. The earlier regime definitions. Same below n<k, 128≤n≤512, and n>k overlapped at n=128, 256, and 512, so their sums could not add to the integrated total, whatever run they came from; they are replaced throughout the paper by the disjoint partition n<k, k≤n≤512, n>512. Against that partition, and from the single run detailed in Section 4.11, the n<k regime accounts for a 95.1% reduction, while the two larger-*n* regimes account for none (−0.1% and −2.0%, respectively; i.e., ASED is marginally the worse of the two there). The SHR n<k subset sum previously printed in this paragraph, $3.86\times 10^{-2}$, was the integrated total of a second run mistranscribed as a subset sum, an arithmetic impossibility, since it exceeded the integrated total of $3.8256\times 10^{-2}$ in Table 4 and it is withdrawn rather than re-estimated, for the reasons set out in Section 4.11. All of the advantage lies in the undersampled regime, and the large headline figure reflects the fact that absolute errors there are orders of magnitude larger than elsewhere. Both aggregations are quoted together wherever the headline appears in this paper, and neither is presented as the reduction. Which one is appropriate is an application question rather than a statistical one. For a screening loop that spends its entire sample budget at n≤32, the S-box search and initialization-analysis settings that motivate the paper, the summed or averaged figure of 95% measures what is actually gained, because that is where essentially all of the error lives. For a practitioner who applies the estimator across a range of window sizes and asks what accuracy to expect at a typical one, the mean of per-size ratios, 24%, is the relevant summary, because it weights every sample size equally. We quote the summed figure first because the motivating application is the first case, and we state the second immediately alongside it; a reader working with long windows should use the 24% figure, and a reader with n≥k should expect no gain at all.

### 4.5. Sensitivity to Departures from Uniformity

Low MSE under the null is necessary but not sufficient: an estimator calibrated to the null could in principle buy accuracy at the price of insensitivity to deviations, and the constant rule is the limiting case. We therefore measure detection power directly. For each estimator, we calibrate a one-sided test of $H=H_{\max}$ at α=0.05 from 2000 fresh null samples (reject when $\hat{H}$ falls below the empirical 5%-quantile) and evaluate the rejection rate over 1000 samples from alternatives of known entropy: biased bits Pr{X=1}=p∈[0.51,0.70] and doped bytes with one symbol carrying mass $p_{0}\in \left\{2,4,8,16,32,64\right\}/256$ and the rest uniform.

Because each estimator is calibrated to its own empirical null threshold, this comparison measures induced test behavior rather than raw entropy-estimation accuracy under alternatives; the latter is reported separately as MSE against the true entropy. Three findings stand out (Table 6, Figure 5 and Figure 6). First, ASED’s power differs only slightly from SHR’s at every tested alternative, alphabet, and sample size, and ASED dominates LAP for bytes at n≥256: the null-calibrated mixture retains sensitivity at the operating points that matter. Second, the constant control has zero power everywhere, exhibiting the confound concretely and separating ASED from it behaviorally. Third, for bits, all genuine estimators have identical power: for k=2, each classical base estimator is a monotone function of the count imbalance, so equal-α tests share the same rejection region power comparisons, which are only informative for larger alphabets. Monotonicity does not follow automatically for ASED, whose weights vary with the sample, so we verified it exhaustively rather than assuming it: for each *n* in the grid, we evaluated ASED at all n+1 count vectors $\left(y_{0},n-y_{0}\right)$ and confirmed that the output is non-increasing in $|y_{0}-n/2|$, with no violation at any sample size. This is a verified property of the fitted model, not a structural guarantee. The same exhaustive check applied to a deliberately under-trained mapper does exhibit violations, so it should be re-verified for any retrained model. Measured against the true entropy of the alternatives (Table 7), ASED’s MSE is at worst equal to SHR’s near the null and lower at moderate deviations for n=64; only at the largest deviation tested ($p_{0}=64/256$, n=64), where both tests already reject with probability one, is ASED’s MSE inflated relative to SHR’s (0.97 versus 0.83), reflecting null calibration far from its design regime.

The power analysis above covers n≥64, but the MSE gain is concentrated at n≤32, so the objection that ASED might buy accuracy there by becoming nearly constant must be settled in that regime specifically. Table 8 repeats the analysis at n∈{8,16,32} with 4000 null and 2000 alternative replications per cell.

At these sample sizes, every genuine estimator induces essentially the same test. Absence of a significant difference would not by itself establish equivalence, so we ran a separate paired non-inferiority experiment. It uses its own draws, 4000 null and 4000 alternative replications per cell, independent of the 2000 alternative replications behind Table 8 and a fully nested bootstrap (B=4000): each resample draws the null replications in pairs, recomputes the 5% critical value of each estimator from the resampled nulls, then draws the alternative replications in pairs and recomputes both powers, so the intervals incorporate the uncertainty of the empirically calibrated thresholds rather than conditioning on them. The margin δ=0.02 was chosen after inspecting the power estimates, so this is a post hoc rather than pre-specified analysis. Pointwise two-sided 95% intervals for $\Delta ={\mathrm{Power}}_{\mathrm{ASED}}-{\mathrm{Power}}_{\mathrm{SHR}}$ lie strictly above −δ in all nine cells; the largest deficit is −0.0040 (CI [−0.0060,−0.0022] at n=8, $p_{0}=64/256$) and the largest surplus +0.0137 at n=16. Three cells at n=8 have intervals excluding zero, so a small but statistically detectable deficit exists there. Because nine pointwise intervals do not license a simultaneous statement, we also bootstrapped the minimum of Δ across the nine cells: its 2.5% quantile is −0.0065, above −δ, so non-inferiority also holds simultaneously at the 0.02 margin. This is a bootstrap lower percentile for the minimum over these nine pre-defined cells, not a correction transferable to any further set of alternatives. ASED is therefore non-inferior to SHR at the post hoc 0.02 margin at every tested operating point, pointwise and simultaneously; this is non-inferiority, not equality. The constant control again has zero power. Non-inferiority at the 0.02 margin therefore holds in the regime where the MSE gain occurs, which is what the objection required. It is not equality: three operating points at n=8 showed small statistically detectable deficits (largest −0.0040, CI [−0.0060,−0.0022]), all contained within the post hoc non-inferiority margin. Absolute power is low at n=8 for all estimators; detecting a $p_{0}=32/256$ deviation from eight observations is close to impossible, so the practical reading is that ASED gives more concentrated point estimates within the post hoc 0.02 non-inferiority margin, not that it enables detection where none was available. Two qualifications belong with this conclusion rather than after it, because they change how far it can be carried. First, the margin δ=0.02 was selected after inspecting the power estimates; the analysis is post hoc throughout and is described as such in the abstract, the conclusions, and here. Second, the comparison is at the matched nominal level, not at the matched achieved level. Section 4.10.2 measures the achieved Type-I error of the strict convention as 0.016 at n=8 and 0.048 at n=32 and shows that the tie rule alone moves reported power by 0.281 at n=8 and 0.094 at n=16, an ambiguity an order of magnitude larger than δ itself. The non-inferiority statement at n=8 and n=16 is therefore exploratory and is not offered as a finding; at n≥32, where the tie-induced gap falls to 0.025 and below and the achieved level is close to nominal, it stands as reported. Restoring exact size control at the two smallest sizes requires a randomized or otherwise exact test or a comparison performed at matched achieved level rather than matched nominal level. We have implemented neither, and in their absence, we do not claim non-inferiority at n=8 and n=16.

Third, Table 8 and Table 9 disagree at n=16, and the resolution is not that one of them is mistaken. At $p_{0}=16/256$, the first reports 0.10 for ASED against 0.09 for SHR; the second reports 0.082 against 0.140: the ordering reverses, and the gap exceeds the binomial half-width at 4000 replications. We examined the null distribution directly rather than adjudicating between the two runs, and the discrepancy turns out to be a property of the testing problem at this sample size, not of either experiment.

Every estimator here is label-invariant, hence a function of the frequency-of-frequencies vector (Lemma 1), and at k=256, that vector takes very few values when n≪k. Enumerating the null distribution from 200,000 draws, the ASED and SHR statistics have 7 atoms at n=8 and 13 at n=16, with *identical* atom probabilities in both cases: the two statistics are strictly monotone relabelings of one another on the null support, so they induce the same family of tests, and the true difference in power at any achievable level is exactly zero. At n=32, the atom-probability multisets no longer coincide, and the two statistics separate genuinely. What differs at n≤16 is only the spacing of the atom *values*, ASED’s cluster within 0.006 bits of each other while SHR’s spread over 0.4 bits, so linear interpolation of the empirical 5% quantile lands on opposite sides of the same atom for the two estimators. Repeating the 4000-draw calibration 200 times, each estimator’s reported power at n=16 takes one of exactly two values, 0.091 or about 0.14, according to which side the interpolated cutoff falls; both tables are sampling that dichotomy.

The underlying reason is that no test of size 0.05 exists at these sample sizes. The achievable levels bracketing 0.05 are 0.0037 and 0.1047 at n=8 and 0.0134 and 0.0713 at n=16; at n=32, they are 0.0480 and 0.1039, so the nominal level is essentially attainable there, and from n=64, the bracket closes to 0.0432 and 0.0531. This is the same discreteness that Section 4.10.2 measures through the tie convention, seen from the side of the critical value rather than the rejection rule, and it is why a randomized or exact test is the only way to compare power at n≤16. We therefore do not reconcile the two tables and do not claim a power difference in either direction at n=8 or n=16: none is identifiable there. The non-inferiority conclusion stands as reported at n≥32, where the tests genuinely differ and the nominal level is attainable. One consequence should be recorded against Table 9: the explanation offered after it, that SHR separates from the $\chi^{2}$, G, and ASED columns at n=16 because its shrinkage intensity is a different function of the counts, is incorrect as stated for SHR, whose null distribution is identical to ASED’s at that size; SHR separates there through the placement of the interpolated critical value alone.

#### Comparison with a Direct Uniformity Test

Comparisons so far are among entropy estimators; because the null is known exactly here, a fair test of whether an adaptive entropy estimator is a useful uniformity diagnostic at all requires comparing it against a direct goodness-of-fit test rather than only against other entropy estimators. As an additional, independently coded check (Section 4.10.3), we calibrated a simulation-exact Pearson chi-square statistic and a likelihood-ratio (G) statistic (both against the empirical null distribution rather than the asymptotic $\chi^{2}$ approximation, which is unreliable when most cells are sparsely populated) alongside ASED and SHR, all at nominal α=0.05, and evaluated power on the same doped-byte alternatives used above. All four detectors see the *same* alternative draws, so differences are assessed by McNemar’s exact test on the discordant pairs rather than by comparing independent rejection rates. Table 9 reports the result.

The comparison does not favor either family uniformly, and the direction depends on both the sample size and which entropy estimator is considered. At n=8 and n=16, the entropy-based detectors are at least as powerful as the direct tests at every tested alternative, significantly so in most cells. At n=32 and the smallest tested deviation ($p_{0}=16/256$), the ordering separates: the G-test (0.248) significantly outperforms ASED (0.220, McNemar $p<10^{-4}$) but is statistically indistinguishable from SHR (0.259, p=0.0008 in SHR’s favor). The honest summary is therefore narrower than a claim of general superiority: the entropy-based approach is not dominated by a classical direct test at these sample sizes, but ASED specifically loses to the G-test at n=32 on the smallest deviation, and where the entropy-based family retains an advantage at that sample size, it is SHR, not ASED, that carries it.

One feature of Table 9 deserves comment because it is easy to misread as an error. At n=16, the $\chi^{2}$, G, and ASED columns are numerically identical across all three alternatives. This is not a transcription mistake: at n=16 with k=256, essentially, every sample consists of singletons and at most a few doubletons, so all three statistics are, over the realized sample space, monotone functions of the number of distinct symbols observed, which takes only five values. The three tests therefore induce the same rejection region and cannot differ. SHR separates from them because its shrinkage intensity is a different function of the same counts. That last sentence is wrong and we correct it here rather than delete it, since the corrected version is the more interesting statement. SHR’s null distribution at n=16 has the same 13 atoms with the same probabilities as ASED’s, so the two are monotone relabelings of one another and cannot induce different tests at any achievable level. SHR separates in this table only because its atom values are spread over roughly 0.4 bits, whereas ASED’s cluster within 0.006, so the interpolated empirical 5% quantile falls on the other side of the same atom. Section 4.5 sets out the consequence: at n≤16, no power difference between these two detectors is identifiable. This degeneracy is a property of the n≪k regime rather than of any particular estimator, and it is the same phenomenon that makes the tie convention of Section 4.10.2 material.

### 4.6. Generalization to Unseen Configurations

To test interpolation beyond the training grid, we retrain the mapper using only the even-indexed sample sizes {8,32,128,512,2048,8192} and evaluate on the held-out odd-indexed sizes {16,64,256,1024,4096,16384}; separately, we evaluate the full-grid model on the alphabet k=16, never seen in training. Table 10 shows that the advantage persists in both cases: on held-out sizes, ASED attains 0.0014 against 0.0075 for SHR (5.4×), and on the unseen alphabet, 0.0195 against 0.0337 (1.7×). The smaller margin at k=16 is expected; the regime descriptor was fit to the geometry of k∈{2,256}, but the direction of the effect is preserved.

To test interpolation across alphabets directly, we retrained the mapper on k∈{2,16,64,256} (400 replications per configuration) and evaluated it on the held-out alphabets k∈{32,128}, alongside the two-alphabet model of the main protocol (Table 11). Both models interpolate: on k=128, they are indistinguishable (3.5 versus $3.6\times 10^{-3}$), and both improve on SHR by an order of magnitude, while on k=32, the multi-alphabet model is 14% better. All of these are interpolation tests, since the evaluated alphabets lie between the trained ones. Extrapolating outside the trained range gives a sharper picture: applying the main model unchanged to k=512 and k=1024 gives integrated MSE $8\times 10^{-4}$ and $7\times 10^{-4}$ against $3.0\times 10^{-2}$ and $2.2\times 10^{-2}$ for SHR (36× and 31×), while at k=4 and k=8, the advantage almost disappears (1.02× and 1.14×). Upward extrapolation was successful at the two tested alphabet sizes, whereas the gain nearly vanished at k=4 and k=8, consistent with the small-alphabet degeneracy discussed for k=2; two sizes do not establish that upward extrapolation is safe in general. Training on four alphabets buys little for interpolation at intermediate *k*, which indicates that the descriptor already transports across alphabet sizes rather than memorizing the two seen in training; it does cost slightly at k=2, where diluting the training set weakens the degenerate small-alphabet case discussed above.

### 4.7. Ablation: What Does the Descriptor Contribute?

A natural objection is that the network might merely have learned the sample size. We test this by retraining the mapper under the same protocol with progressively poorer inputs: a constant input (a single fixed global mixture), the ratio n/k alone, the five-dimensional regime summary of Equation (3), and the full 37-dimensional descriptor; Table 12 reports byte-sequence integrated MSE on a common evaluation stream.

The result is sharper than the objection anticipates. Neither a fixed global mixture nor weights conditioned on n/k alone improve materially on SHR in integrated MSE. The reason is regime-specific and must be stated carefully. At n=8, where condition (8) is satisfied and certified by the bootstrap, no per-regime convex combination of the six estimators can improve on the best member, so knowing the regime is not enough. At n=16, the plug-in estimate of ${\bar{M}}_{k,n}$ satisfies the condition, but the bootstrap certification is inconclusive, so the same conclusion is supported by the point estimate only. At n=32, the condition fails, and a fixed mixture *does* improve on SHR by 17%, as computed above, yet it remains a factor of three worse than ASED ($1.85\times 10^{-3}$ versus $6.5\times 10^{-4}$). The integrated-MSE figures for the n/k-only mapper therefore do not by themselves establish that every per-regime mixture is useless; the per-regime computation does, at the sizes where the condition holds, and quantifies the residual gap where it does not. Essentially the entire gain appears once the weights can respond to the *per-sample* coverage statistics $\left(C,f_{1}/n,f_{2}/n,\hat{s}\right)$, which predict the plug-in error of the individual sample; the FoF tail adds no measurable improvement for the i.i.d. alternatives studied here. This localizes the mechanism with precisely sample-conditional bias correction driven by coverage features and implies that a five-feature “ASED-lite” suffices in the i.i.d. setting, while the full descriptor is retained because it may provide headroom for structured deviations (Markov, periodic), whose signature would appear in the FoF tail; whether it does is an empirical question left open here.

### 4.8. Comparison with General-Purpose Estimators

The base set is deliberately restricted to closed-form estimators, but the comparison should not be. Table 13 adds two further general-purpose estimators evaluated on a common stream: the Chao–Wang–Jost estimator CWJ [20] and the Grassberger correction GR [13] alongside NSB, which is also reported under the full 1000-replication protocol in Table 4 and Table 5.

Our NSB implementation was validated before use: on three distributions of known entropy at k=256 (uniform, Zipf s=1, and a half-mass two-point mixture), its bias at n=256 is −0.10, −0.09, and +0.10 bits against −0.83, −0.63, and −0.71 for the plug-in, and the estimate is stable to four decimals as the integration grid is refined from 20 to 200 points (7.8962, 7.8951, 7.8946). It therefore behaves as documented; its poor showing below is a property of the uniform-null criterion, not of the implementation. NSB, CWJ, and GR are not competitive under the uniform null, and the reason is structural rather than incidental: they are built to be accurate for *arbitrary* distributions and therefore cannot exploit the uniform target that SHR shrinks toward and that ASED is calibrated on. The comparison is thus informative in one direction only; it confirms that the gain is specific to the uniformity-diagnostic setting, and Section 4.9 examines the converse regime, where the ordering reverses. We also implemented a simplified linear-programming reconstruction of the Unseen estimator [10]; it was not competitive in extreme undersampling (integrated MSE two orders of magnitude above the others, with estimates exceeding $H_{\max}$ at n=8). We attribute this to our reconstruction rather than to the method and therefore exclude it from Table 13. The BUB estimator of Paninski [8] is omitted for the same reason: its published behavior depends on a tuning parameter and on a linear-programming construction whose released implementations we could not reproduce to the accuracy required for a fair comparison at these sample sizes, and reporting a self-coded approximation under our protocol would misrepresent it. A faithful comparison against the authors’ implementation remains open.

### 4.9. Beyond the Null: Strongly Non-Uniform and Dependent Sources

The power analysis probes alternatives close to the null. We now go deliberately far from it to delimit where ASED must not be used. Table 14 reports MSE against the true entropy for three families, specified here in full. *Zipf*: $p_{i}=i^{-s}/\sum_{r=1}^{k}r^{-s}$ for i=1,…,k with s∈{0.3,0.6,1.0}, sampled i.i.d. *Markov*: a first-order chain on K with $P_{ii}=p_{\mathrm{stay}}$ and $P_{ij}=\left(1-p_{\mathrm{stay}}\right)/\left(k-1\right)$ for i≠j, and $p_{\mathrm{stay}}\in \left\{0.5,0.9\right\}$; the initial state is uniform, which is also the stationary distribution of this doubly stochastic kernel, so the *marginal* entropy is exactly 8 bits at every position while the sequence is strongly dependent. *Periodic*: a phase *u* is drawn uniformly from {0,…,63}, and the window is $x_{t}=\left(u+t\right)\;\mathrm{mod}\;64$ for t=0,…,n−1, the 64 attainable values being regarded as a subset of the 256-symbol alphabet. Each position then has the same *time-marginal symbol distribution* under a uniformly random phase uniform on those 64 symbols, so $H\left(X_{t}\right)=\log_{2}64=6$ bits exactly, and for *n*, a multiple of 64, each symbol occurs exactly n/64 times in the window. Drawing the phase from the full alphabet instead would make every positional marginal uniform on 256 symbols and the target 8 bits while leaving the within-window count pattern unchanged; we use the former so that the tabulated target matches the quantity the estimators are asked to recover. No block permutation is applied. Note that this is the time-marginal symbol entropy under a random phase, not the entropy rate: a deterministic periodic sequence has entropy rate zero, and neither $H(X_{1},\ldots ,X_{n})$ nor $\lim_{n}n^{-1}H\left(X_{1},\ldots ,X_{n}\right)$ is the target here.

The picture is clear and partly unfavorable to ASED, which is why we report it. Near the null mild Zipf skew, where the true entropy is within a few hundredths of $H_{\max}$, ASED matches the best estimator. Far from the null, it fails: at s=1.0, its MSE is 29× that of NSB and 16× that of SHR, and on a periodic source, it is worse than both. The failure is exactly the one predicted by the scope statement: a mixture whose weights are calibrated where $H\approx H_{\max}$ inherits the behavior of its Bayesian-smoother components when the sample is strongly non-uniform, and those components are the wrong ones there. ASED is a uniformity diagnostic, not a general-purpose entropy estimator, and Table 14 is the quantitative statement of that boundary. One result runs the other way: under strong dependence with a uniform marginal, ASED and LAP estimate the marginal entropy an order of magnitude more accurately than SHR or NSB, because the FoF profile of a dependent sample resembles that of a smaller effective sample. We note, however, that no marginal-entropy estimator detects the dependence itself: the marginal is genuinely uniform, so this regime falls outside what any estimator in this comparison is designed to  diagnose.

#### 4.9.1. Is Uniform-Only Training the Cause?

To separate the effect of the training distribution from the effect of the base set, we retrained the mapper on a 50/50 mixture of uniform samples and near-uniform perturbations (one symbol carrying mass $p_{0}\in \left[1,4\right]/k$), with the true entropy of each source as the target. The retrained mapper is indistinguishable from the original under the null (integrated MSE 0.00177 versus 0.00171) and improves only marginally far from it (Zipf s=1.0: 1.054 versus 1.178; Zipf s=0.6: 0.027 versus 0.030). Broadening the training distribution over near-uniform sources is therefore *not* what limits performance on strongly skewed sources; the results indicate that the convex hull of the current base set is the principal observed limitation, its members being themselves inaccurate in exactly the regime in which Proposition 1 becomes nearly saturated. Extending ASED beyond uniformity diagnostics would likely require adding general-purpose members such as NSB to the base set, not merely retraining on a wider family. The general question of what happens under an arbitrary family of source distributions has a partial answer in three parts and one genuine gap. Proposition 1 gives the distribution-free safety upper bound: for any source, the finite-sample inequality holds, and consistency and convergence rates are inherited whenever every member of the base set has the corresponding property under that source. Table 14 gives the empirical ceiling for six structured families, and the retraining experiment above indicates that, under the training distributions and optimization variants evaluated, the current base set is the primary observed constraint rather than the training measure, without establishing it as the unique limiting factor. What is missing is a characterization of the *transfer* regime: for which perturbation families P around the uniform does a mapper fitted under P∈P retain the oracle behavior of Theorem 1 at Q∉P? Since the theorem is stated conditionally on φ, the natural route is a bound in terms of how much the conditional error geometry M(φ) moves between *P* and *Q*, which we leave open.

#### 4.9.2. Why a Neural Mapper?

The weight function need not be a network, and the objective admits two distinct strategies: fitting the weights directly against the mixture loss of Equation (5) or fitting a surrogate that predicts each base estimator’s squared error from φ and converting those predictions into weights. Table 15 evaluates six families under both strategies on the byte protocol.

The honest reading is that the architecture is not what carries the result. Every sufficiently flexible family evaluated, except the linear-softmax and Nadaraya–Watson specifications, lands within a factor of two of the MLP and an order of magnitude below the best fixed estimator; gradient boosting with hard selection is in fact marginally better ($1.54\times 10^{-3}$). Only the two genuinely restrictive families fall behind: a linear softmax, which cannot express the non-linear dependence, and kernel regression, which degrades because the FoF features are highly non-uniformly distributed across regimes. We retain the MLP because it optimizes the deployment objective directly, produces smooth weights, and costs nothing at inference, not because it dominates. We can now be precise about the comparison rather than merely cautious about it. Using the independent implementation of Section 4.10.3, we fitted all three candidates, the full 37-feature mapper, the five-feature ASED-lite variant, and the gradient-boosted surrogate with hard selection, on a common training stream and evaluated them on a single locked evaluation stream never used in fitting, then bootstrapped the differences by resampling the replication index so that all three see the same resamples. Table 16 reports the result. *None of the three pairwise differences exclude zero.* The gradient-boosted selector has the lowest point estimate, as seen in Table 15, but the paired interval for its advantage over the full mapper spans zero, as does the interval for ASED-lite against either alternative. No statistically supported ordering among the three families is established by these data, and none should be inferred from the point estimates alone; a practitioner may choose among them on grounds of interpretability, inference cost, or implementation convenience without accepting an accuracy penalty that these experiments can detect.

#### 4.9.3. Why 37 Features?

The tail length was fixed a priori to cover FoF coordinates up to i=34. Retraining with truncated descriptors of dimension 5, 13, 21, and 37 gives integrated MSE 0.00173, 0.00170, 0.00170, and 0.00171, respectively: the choice is not load-bearing for i.i.d. sources, consistent with the ablation of Section 4.7. We report the full descriptor because it is the natural occupancy-profile object and leaves room for structured alternatives, while noting that the five-feature variant matches the integrated-MSE performance of the full descriptor under the evaluated i.i.d. protocols, with no measurable gain from the FoF tail. For the domain validated here, we therefore recommend ASED-lite as the parsimonious implementation, treating the 37-dimensional descriptor as an extensible architecture whose extra coordinates are motivated by, but not yet demonstrated on, structured alternatives. Read together with Table 16, this settles what the contribution of the paper is and is not. Of the three weight-function families compared on a common locked stream, the full 37-feature mapper, the five-feature variant, and a gradient-boosted surrogate with hard selection, no pairwise difference has a bootstrap interval excluding zero, and the surrogate has the lowest point estimate. Neither the MLP nor the 37-dimensional descriptor is therefore load-bearing, and we claim neither as a contribution. What the experiments support is the more general proposition: convex mixtures of classical entropy estimators whose weights are conditioned on per-sample occupancy statistics outperform any fixed member of the mixture in the undersampled regime under a uniform null, essentially independently of the function class used to produce the weights. The MLP over 37 features is the realization we fitted and report in full; ASED-lite is the implementation we recommend, and a practitioner preferring gradient boosting may use it without an accuracy penalty these experiments can  detect.

### 4.10. Cryptographic and Defective Sources

All experiments so far use parametric families. Because the paper motivates the problem by cryptographic practice, we close the loop on actual generator output. We evaluate five sources at n∈{32,128,512} with 400 independent windows each, every window using an independent seed. (i) A reference stream generated by SHA-256 in counter mode: with a fresh 32-byte key κ per window, successive output blocks are $\mathrm{SHA}256(\kappa \;∥\;{\mathrm{BE}}_{64}\left(c\right))$ for c=0,1,2,…, concatenated and truncated to *n* bytes, where ${\mathrm{BE}}_{64}$ is the 64-bit big-endian encoding, and ‖ is byte concatenation. (ii) An RC4 keystream from a fresh 16-byte key, discarding the first 768 output bytes. (iii) RC4 initial output from a fresh 16-byte key with no discard. (iv) A truncated linear congruential generator $x_{t+1}=\left(1103515245\;x_{t}+12345\right)\;\mathrm{mod}\;2^{31}$ with a fresh uniform seed, emitting the byte $(x_{t+1}\gg 24)\wedge 0\mathrm{xFF}$. (v) A source with controlled marginal bias $p_{0}=8/256$ on one symbol and the remaining mass uniform.

Three observations follow. Of the three cryptographic streams in Table 17, we treat SHA-256 in counter mode and RC4 after discarding 768 bytes as the references without known defects under this protocol, with the initial output of RC4 analyzed separately below. For these two, ASED is closer to $H_{\max}$ than SHR at n=32 and identical from n=128, matching the synthetic uniform results and providing evidence that the uniform-null behavior transfers to the two reference streams under the tested protocol. The truncated LCG deserves a precise statement, because its marginal entropy is available in closed form. The multiplier 1103515245 is odd, so $\gcd(1103515245,2^{31})=1$ and the affine update $x\mapsto 1103515245\;x+12345\;\mathrm{mod}\;2^{31}$ is a bijection on $Z/2^{31}Z$; under a uniform seed, every state $x_{t}$ is therefore exactly uniform. The emitted byte is $(x_{t}\gg 24)\wedge 0\mathrm{xFF}$, and since $x_{t}<2^{31}$, the shift leaves only $2^{31-24}=128$ attainable values, each with exactly $2^{24}$ preimages. The one-byte marginal is thus exactly uniform on a support of half the nominal alphabet, giving $H=\log_{2}128=7$ bits, exactly a genuine marginal deficit of one bit relative to $H_{\max}=8$, caused by the truncation discarding the alphabet rather than by serial dependence. A direct computation over $4\times 10^{5}$ uniform seeds returns 6.99977, matching 7 minus the Miller–Madow bias (K−1)/(2Nln2) for K=128. Read against this target, both estimators are *above* the true value at every window length and descend towards it as *n* grows (7.55 at n=512), which is the expected behavior of null-calibrated estimators on a source whose support is half the assumed alphabet. Both estimators therefore register a growing deficit relative to $H_{\max}$, with ASED slightly higher at short windows. Because this source lies under the alternative H=7<8, failing to register the deficit is not the avoidance of a false alarm but a loss of detection: its upward null-calibration bias makes the diagnostic conservative at short windows and reduces sensitivity to this genuine one-bit marginal-entropy deficit. The overestimation is substantial, with mean estimates of about 7.96 at n=32 and 7.55 at n=512 against a true 7, so a support-restricted alternative of this kind is detected only slowly as the window grows. Third, pooling successive output positions within a window does not resolve position-specific structure, and RC4 is the standard example.

#### 4.10.1. Position-Conditional Analysis of Early RC4 Output

Early RC4 keystream carries well-documented *position-specific marginal* biases, notably that of the second output byte toward zero [34,35]. A pooled-window entropy cannot resolve them, because averaging over positions dilutes each one; the comparison above is therefore silent about these biases rather than evidence against their detectability. The correct protocol conditions on the output position. We generated 200,000 independent 16-byte keys, recorded the first sixteen keystream bytes of each, and analyzed the marginal distribution of $Z_{t}$ across keys separately for each position *t*. The reference computation over all 200,000 keys reproduces the known effect: $\mathrm{Pr}\{Z_{2}=0\}=0.0079$ against the uniform 0.0039, a factor of two as predicted, while positions t≠2 give 0.0037–0.0040. The entropic consequence is nevertheless small: position-wise marginal entropies are 7.99668 bits at t=2 against 7.9989–7.9991 elsewhere, a deficit near 0.002 bits. Estimating the entropy of $Z_{2}$ from *n* independent keys, neither ASED nor SHR detect the anomaly at the sizes of interest: rejection rates at α=0.05 are 0.06, 0.05, and 0.09 for n=64,256,1024 (1000 replications, so binomial half-widths near ±0.018), close to the nominal size and identical for the two estimators; the value at n=1024 is nominally above 0.05 but corresponds to very low power in any case.

The conclusion is sharper than a pooled design would license. The position-conditional design does resolve the distributional bias, and it is now possible to say precisely how much sharper. As an additional check using the independent implementation of Section 4.10.3 (not the primary pipeline behind the results above), we compared three detectors under identical null calibration and the same resampled draws from the reference $Z_{2}$ pool: ASED, SHR, and a direct test on the count of $Z_{2}=0$ occurrences among *n* keys, calibrated by simulation against the exact null binomial. Table 18 reports rejection rates with 95% Wilson intervals. The direct test dominates throughout: at n=500, it already has 42.5% power against 6.1% for ASED and SHR combined, and it reaches near-certain detection by n=5000, roughly an order of magnitude earlier in sample size than the entropy-based estimators. This confirms directly what was previously only a plausible reading: *a test targeting $\mathrm{Pr}\{Z_{2}=0\}$ is the right instrument for this specific bias*, and the entropy-based estimators, while correctly calibrated in general, are the wrong tool for it. Other purpose-built statistics beyond this one direct test were not evaluated, so the comparison is between three specific detectors rather than an exhaustive search over possible tests; it nonetheless illustrates the scope limit stated throughout with a concrete, quantified alternative rather than a speculative one.

Other entropy estimators and purpose-built statistics beyond the one direct test above were not evaluated, so this is an observation about the three detectors tested rather than an exhaustive impossibility result for entropy-based detection more generally; it nonetheless illustrates the scope limit stated throughout, now with a quantified alternative rather than a speculative one.

#### 4.10.2. Tie-Breaking at Empirical Critical Values and Its Consequences

Every test in this paper calibrates its critical value as an empirical quantile of a simulated null distribution. When that null distribution is highly discrete, as it is throughout the n≪k regime that motivates the paper, a non-negligible probability mass sits *exactly at* the critical value, and the rule for handling it stops being a rounding detail. We state the convention explicitly and quantify its cost because the effect is larger than we anticipated.

The convention used throughout this paper is *strict*: the null is rejected only when the statistic falls strictly below (for entropy estimators) or strictly above (for $\chi^{2}$ and G) the empirical critical value, with ties resolved in favor of the null. Table 19 shows why the choice matters. At n=8, the ASED null distribution takes only four distinct values over 8000 draws, and roughly 10% of the null mass sits exactly at the 5% quantile; switching from the strict to the inclusive convention moves reported power at a fixed alternative from 0.374 to 0.655, a swing of 28 percentage points attributable entirely to the tie rule.

The consequence is more serious than a loss of precision, and we report it rather than leave it implicit. Because the mass at the critical value is substantial, *neither* convention attains the nominal level at the smallest sample sizes. Measured on fresh null draws at k=256, the strict convention gives an achieved Type-I error of 0.016 at n=8 and 0.048 at n=32, while the inclusive convention gives 0.108 and 0.102, respectively; nominal α=0.05 is attained by neither at n=8, the strict rule being conservative and the inclusive rule anti-conservative by more than a factor of two. Exact size control with a discrete statistic would require a randomized test, which we have not implemented.

Two consequences follow for how the power comparisons in this paper should be read. First, the small-sample power figures in Table 8 and Table 9 are comparisons at the matched *nominal* level, not at the matched achieved level, and at n=8, the achieved level is well below the nominal level for all detectors under the strict convention. Second, and more importantly for the non-inferiority analysis of Section 4.5, the δ=0.02 margin is of the same order as the tie-induced ambiguity at n=8 and n=16; the non-inferiority conclusion at those two sample sizes should therefore be regarded as resting on a convention rather than as a sharp finding, which reinforces rather than softens the existing description of that analysis as post hoc and exploratory. The conclusion at n=32 and above, where the gap falls to 0.025 and below, is not affected in the same way.

#### 4.10.3. Independent Verification of the Exploratory Pipeline: The Locked Confirmatory Evaluation

Section 3 discloses that the design choices behind the primary pipeline, the feature map, architecture, and loss weighting were made while observing results on the same evaluation protocol used to report them, so no independent model-selection split separates development from the assessment reported in Section 4.1, Section 4.2, Section 4.3, Section 4.4, Section 4.5, Section 4.6, Section 4.7, Section 4.8, Section 4.9 and Section 4.10. That limitation is not resolved by what follows. As a partial, external check on whether the headline result is nonetheless reproducible under a genuinely locked protocol, we built a separate implementation, its own code, its own reconstruction of the 37-dimensional feature descriptor from the description in Section 3 rather than a copy of the descriptor actually used, and its own weight-mapper training run, and evaluated it under a design in which the training, validation, and test data are drawn from three independent random-seed streams that are never mixed. The architecture (two hidden layers of width 64 over the 37-dimensional input, six-way softmax output) was fixed in advance to match Table 1; this yields exactly 6848 weights and 134 biases, matching the primary model’s parameter count exactly and giving some confidence that the reconstruction is structurally faithful. The test stream was evaluated once, at the end, and used for nothing else. This independent implementation, together with the auxiliary scripts for the RC4 $Z_{2}$ analysis, the Zipf sweep, the support-restricted generator, and the fresh-null power calibration, is released as Supplementary Materials (Code S2).

Under this protocol, the independent implementation reaches an integrated MSE of $1.744\times 10^{-3}$ for ASED against $3.564\times 10^{-2}$ for SHR at k=256, a 95.1% reduction, closely matching the primary pipeline’s reported $1.746\times 10^{-3}$ against $3.83\times 10^{-2}$ and 95.4% reduction. Table 20 reports the full per-size breakdown, along with the integrated totals and the internal-consistency checks that follow. Four further qualitative findings from the primary pipeline were checked in the same independent run without being specifically targeted for reproduction. The k=2 instability described in Section 3 reproduced as elevated validation-loss variance during training. The support-restricted overestimation of Section 4.10 reproduced closely (7.96 at n=32 and 7.55 at n=512 against the primary pipeline’s 7.96 and 7.55). The Zipf degradation of Section 4.9 was reproduced in direction and rough magnitude but not exactly (12.7× against NSB at s=1.0, versus the primary pipeline’s 29×, using an independently implemented and separately validated NSB estimator that runs at roughly 55–60% of the primary implementation’s reference bias scale). Finally, the covariance condition of Corollary 1 was re-verified by directly solving $\min_{\lambda \in \Delta^{M}}\lambda^{⊤}M\lambda$ with a constrained quadratic program (confirmed to global optimality: 150 independent random restarts across 10 batches converged to the same solution to within $10^{-18}$, as guaranteed by *M*’s positive semi-definiteness) on an independently estimated error covariance matrix at n=8,16,32,64. The qualitative pattern reproduced exactly: the condition holds at n=8,16,64 (the best single estimator is the unique optimal fixed mixture, 0% improvement available) and fails at n=32, where the optimal fixed mixture places weight (0.717,0.283) on (SHR, LAP) and improves on the best single estimator by 12.5%, the same qualitative finding as the primary pipeline’s (0.66,0.34) weighting and 17% improvement, on an independently estimated covariance matrix.

Table 20 is internally consistent by construction, and we note the checks explicitly because their absence in the primary run is the subject of Section 4.11: the twelve per-size values sum to the reported integrated total; the n<k subset sum ($1.694\times 10^{-3}$ for ASED against $3.559\times 10^{-2}$ for SHR) is strictly smaller than that total, accounting for 97.1% and 99.9% of it, respectively; and the paired difference of the integrated values, $-3.389\times 10^{-2}$, follows arithmetically from the two columns. The per-size breakdown also shows where the aggregate advantage comes from and where it does not: the reduction is 99.8% at n=8 and falls monotonically to approximately zero by n=128, beyond which ASED and SHR are indistinguishable and ASED is marginally worse in several cells. This is the same regime-concentration reported for the primary pipeline in Section 4.4 and is the reason the headline figure is so sensitive to the aggregation scheme.

This exercise has clear limits, stated plainly. The feature descriptor is a reconstruction from the manuscript’s own prose description, not a byte-for-byte copy of the code behind Section 4; a single training run is reported, without restart variance; and the NSB comparison used to check the Zipf result is calibrated to a different scale than the primary implementation’s, so the 12.7× figure should be read as directionally, not numerically, confirmatory. Most importantly, this is external corroboration by a second, independently written codebase, not a resolution of the primary pipeline’s own model-selection contamination: the results reported in Section 4.1, Section 4.2, Section 4.3, Section 4.4, Section 4.5, Section 4.6, Section 4.7, Section 4.8, Section 4.9 and Section 4.10 still lack an independent split of their own, and closing that gap, rather than corroborating it externally, remains the priority stated in Section 3.

Finally, we record the treatment of the feature standardization constants μ and ς of Equation (4), since their status was previously left implicit. They are estimated once, from the first training batch of a given run, and held fixed thereafter: they are not recomputed at validation or test time, and each independently trained model, including each retraining in Section 4.6 and Section 4.9, and the verification model of this section, carries its own set. Reusing one model’s constants with another model’s weights is therefore not valid. For the verification model, all 37 pairs for each of k=256 and k=2 accompany the released coefficients. We note one respect in which the verification model is not a substitute for the primary one on this point: its descriptor is a reconstruction from the prose of Section 3 and does not coincide coordinate-for-coordinate with Equation (3), so its constants document the verification pipeline rather than the primary one, whose corresponding values must come from the primary implementation.

### 4.11. Diagnosis and Correction of a Numerical Inconsistency in the Exploratory Run

Two related numerical inconsistencies were identified in review, and we can now state their common cause precisely rather than merely acknowledging them. Section 4.4 reports the n<k regime-sum for SHR as $3.86\times 10^{-2}$, which exceeds the integrated total of $3.8256\times 10^{-2}$ given in Table 4, impossible for a subset of a sum of non-negative terms. Separately, the paired difference reported in Section 3 is $-3.69\times 10^{-2}$, whereas the full-precision integrated values in Table 4 imply $-3.651\times 10^{-2}$.

Both arise from the same defect: two distinct evaluation runs were used within a single section. The main tables derive from one run, giving integrated values $1.7462\times 10^{-3}$ (ASED) and $3.8256\times 10^{-2}$ (SHR); the paired randomization analysis derives from a second run, giving $1.6905\times 10^{-3}$ and $3.8601\times 10^{-2}$. The second run is internally consistent; its stored paired difference agrees with the difference of its own integrated values to twelve decimal places, and it is the source of the $-3.69\times 10^{-2}$ figure, which is correct *for that run* but not for the tabulated one. The $3.86\times 10^{-2}$ figure in Section 4.4 is the same second run’s *integrated* SHR value, reported there as though it were the n<k subset sum; the ratio is exactly 1.000, confirming it is the total rather than a subset. The ASED figure printed alongside it, $1.64\times 10^{-3}$, is by contrast a genuine n<k subset of that run’s total, at 97.0% of it, closely matching the 97.1% subset share measured independently in Section 4.10.3. The defect is therefore confined to a single transcribed quantity, not to the underlying computation.

A third defect, independent of the first two, concerns the regime definitions themselves. The three regimes named in Section 4.4, n<k, 128≤n≤512, and n>k are not a partition of the twelve-point grid: n=128 falls in the first two, and n=256 and n=512 fall in the last two. Sums reported against these definitions therefore cannot add to the integrated total, whatever run they come from, since three of the twelve sizes are counted twice. A disjoint definition is required; we use n<k, k≤n≤512, and n>512 below.

To establish what an internally consistent regeneration looks like, and to give a template against which the primary run can be checked, we regenerated the complete byte-sequence evaluation from a single run of the independent implementation of Section 4.10.3 (1000 replications per size, one fixed seed, disjoint regimes), with the consistency conditions enforced as assertions rather than checked afterward. Table 21 reports it. Every aggregate follows from the per-size column: the three regime sums add to the integrated total exactly; each subset is strictly below its total; the paired difference equals the difference of the integrated values; and all four aggregation schemes are computed from the same per-size values. The four schemes give reductions of 94.94% (summed), 94.94% (arithmetic mean), 92.11% (log-trapezoidal), and 23.15% (mean of per-size ratios), reproducing the qualitative spread reported in Section 4.4 from a source in which the spread can be verified. The paired difference is $-3.416\times 10^{-2}$ with a 95% bootstrap interval of $\left[-4.335\times 10^{-2},-2.666\times 10^{-2}\right]$, and a sign-flip randomization test over 20,000 assignments yields no assignment as extreme as the observed statistic.

We are explicit about what this table is and about the status of the affected figures in the present version. It is an internally consistent evaluation produced by the independent implementation, not a re-run of the exploratory pipeline: the feature descriptor is a reconstruction, so its values differ from the exploratory run’s in the third significant figure, and it does not license substituting these numbers for those of Table 4. What it establishes is that the structure of the result, a reduction above 94% under summed aggregation, falling to roughly 23% under per-size-ratio aggregation, with essentially all of the advantage confined to n<k, survives regeneration under a protocol in which every aggregate is verifiable from its own constituents.

Three corrective actions have been taken in this revision rather than deferred, so that no figure in the manuscript now carries a provisional status. First, the overlapping regime definitions are retired and replaced throughout by the disjoint partition n<k, k≤n≤512, n>512; every regime split reported in the paper uses it. Second, the two quantities transcribed from a second run, the SHR n<k subset sum of $3.86\times 10^{-2}$ and the paired difference of $-3.69\times 10^{-2}$ with its interval, have been *removed* from Section 4 and Section 4.4, not merely annotated. The paired difference is replaced by the value implied by Table 4 itself ($-3.651\times 10^{-2}$) and the interval by the one produced by the single run of this table, attributed to it; the subset sum is replaced by the corresponding figure from this table. Third, the single-run rule stated in Section 4 is applied to every aggregate in the paper, and the aggregation code enforces the consistency conditions as run-time assertions (regime sums against the integrated total, subset sums strictly below their totals, paired differences against the difference of integrated values) rather than checking them afterwards.

The result is that no claim in this manuscript rests on a quantity drawn from a run other than the one behind its own table, and the word “provisional” no longer applies to any reported figure. One item remains genuinely outstanding, and we state it rather than conceal it: the exploratory pipeline of Section 4 has not itself been re-executed under the assertion-checked procedure, so the third significant figure of Table 4 may move when it is. Nothing in the paper’s conclusions depends on that digit. The locked confirmatory evaluation of Section 4.10.3, which was produced under exactly that procedure by an independently written implementation, reaches the same conclusion on its own evidence (95.1% against 95.4%, with the same regime concentration), and it is the evaluation on which the reader is asked to rely.

## 5. Discussion

The central finding is that a learned, sample-conditional mixture of classical estimators improves substantially on the best fixed estimator for short cryptographic byte sequences, in the undersampled regime and under a uniform marginal null: a reduction in integrated MSE over SHR of 95.4% under summed or averaged aggregation and 24% under the mean of per-size ratios, confined entirely to n<k and reproduced at 95.1% by the locked confirmatory evaluation of Section 4.10.3, with statistically separated bootstrap intervals, detection power non-inferior at a post hoc 0.02 margin against the near-uniform alternatives tested (confirmatory for n≥32, exploratory at n=8 and n=16; Section 4.10.2), an advantage that survives held-out sample sizes and an unseen alphabet, and an ablation localizing the gain in the per-sample coverage features rather than in knowledge of the regime. Theorem 1 and Corollary 1 supply the matching explanation: the excess risk over the sample-conditional oracle is governed by how well the weights are approximated, and where condition (8) holds, no fixed or regime-indexed mixture of the base set can improve on its best member. Proposition 1 bounds the other side, and its near-saturation on skewed sources (1.170 measured against a bound of 1.184) shows that the failure in Section 4.9 is the degradation the theory predicts rather than an unmodeled pathology.

### 5.1. Why the Three-Layer Evaluation Is the Result

Because the degenerate rule $\hat{H}\equiv \log_{2}k$ attains zero null error by construction (Section 1), low null MSE is not by itself evidence of estimation ability, and convexity does not rule out a constant-valued adaptive combination. The decisive evidence is therefore behavioral: the constant control has zero power everywhere, while ASED’s power is non-inferior to SHR’s at a post hoc 0.02 margin (Section 4.5). The benchmark-gap analysis sharpens the interpretation of what was learned. At n≤32, ASED outperforms every *evaluated* fixed estimator and the per-regime best-single-estimator benchmark, even though the errors of its dominant components are positively correlated; the gain therefore comes from weights that covary with the FoF features predicting the plug-in error, a learned bias correction calibrated to the null. The power analysis bounds the cost of that calibration: it is contained within the post hoc 0.02 non-inferiority margin, although three operating points at n=8 exhibit a small statistically detectable deficit, with a measurable MSE penalty only at gross deviations where every test already rejects with probability one. The ablation also clarifies what the contribution is and is not. Although the full FoF tail does not improve the i.i.d. uniform-null results over the five-feature coverage summary, it is retained because it represents the occupancy-tail extension that may be needed for future structured alternatives; in the present experiments, the effective signal is concentrated in the low-order coverage coordinates, and the method is most accurately described as an interpretable convex mixture of classical estimators whose weights respond to per-sample coverage statistics.

### 5.2. Practical Implications

For frameworks that apply marginal-entropy statistics to short output blocks, ASED offers SHR’s detection behavior with substantially more concentrated point estimates under the null and detection power non-inferior to SHR’s at a 0.02 margin. We say “more concentrated point estimates” rather than “narrower confidence intervals”: lower MSE does not by itself deliver shorter intervals with correct coverage, and we construct no interval procedure here. In S-box design loops that screen thousands of candidates on small output samples [7], the accuracy at n≤32 beyond the per-regime best-single-estimator benchmark may permit earlier or cheaper comparisons between candidates. In stream cipher initialization analysis [3], the held-out results support applying the trained mapper at window sizes between the grid points without retraining. We should not overstate what the generator experiments add to this case, because they are the weakest evidence in the paper for a practical advantage over SHR rather than the strongest. On SHA-256 counter mode and post-discard RC4, the two estimators differ only at n=32, by about 0.003 bits, and coincide from n=128 onward. On the truncated LCG, both overestimate the true 7 bits substantially (≈7.96 at n=32 and 7.55 at n=512), with ASED slightly the worse of the two at short windows. On the RC4 $Z_{2}$ bias, a purpose-built direct test detects the defect roughly an order of magnitude earlier in sample size than either estimator, and the two entropy-based detectors are indistinguishable from each other throughout. The case for preferring ASED to SHR therefore rests on the null-regime MSE at n≤32 and on non-inferior power at n≥32 and not on the cryptographic-source experiments, which show the two behaving very similarly wherever they were directly compared. These statements are confined to *marginal-symbol* Shannon-entropy diagnostics under a uniform marginal null. ASED does not assess cryptographic randomness, unpredictability, independence, or min-entropy, and it is not a certification procedure: Section 4.10 exhibits a source (early RC4 output) whose known position-specific defect went undetected by both estimators tested at realistic sample sizes, and certification remains a min-entropy problem per SP 800-90B [6] and AIS 20/31 [23].

### 5.3. Limitations

First, the theory is open: weights and base estimates are dependent functions of the same FoF vector (Remark 1), so no learning-theoretic oracle guarantee for the fitted mapper follows from the offline construction alone, in contrast to the guarantees available for the super learner [27] or BUB [8]. Second, the neural mapper is a convenience rather than a necessity: Table 15 shows several simpler families within a factor of two and a gradient-boosted surrogate marginally ahead. Third, ASED is not a general-purpose entropy estimator, and Section 4.9 quantifies the boundary: on strongly skewed (Zipf s=1) or periodic sources, its error exceeds that of NSB by more than an order of magnitude, and retraining on a broader near-uniform family does not repair this: under the tested training distributions and optimization variants, the convex hull of the current base set appears to be the principal observed limitation, without being established as the unique limiting factor. The bit-sequence mapper likewise realizes only part of the improvement available in its own base set, leaving MIN ahead; the FoF descriptor is nearly degenerate at k=2. Fourth, the alternatives tested are single-parameter families (biased bits, doped bytes), and ASED is calibrated under i.i.d. sampling: temporal dependence, Markovian artifacts, and block correlations may alter the FoF distribution in ways the current descriptor does not fully capture. Fifth, the headline reduction depends on the aggregation scheme: it is 95.4% for summed or averaged MSE but 24% for the mean of per-size ratios (Section 4.4), and no independent model-selection split separates development from final evaluation. Sixth, the comparison now includes NSB, CWJ, and Grassberger (Section 4.8) but still omits BUB, whose faithful implementation requires the regularized minimax program, and reports no fair figure for Unseen, since our simplified reconstruction was not competitive, and we do not attribute that to the original method. Seventh, the small-sample power comparison is not identifiable: at n≤16, the ASED and SHR statistics take the same few null values up to relabeling, no test of size 0.05 exists, and the power differences reported in Table 8 and Table 9 are artifacts of where an interpolated empirical quantile falls between atoms. The tests reported here are calibrated at nominal rather than achieved level. The null statistic is highly discrete for n≤16, where neither tie convention attains α=0.05, and the choice of convention alone moves reported power by up to 0.281; the resulting ambiguity exceeds the non-inferiority margin, so the power conclusions at the two smallest sample sizes rest on a convention and are exploratory (Section 4.10.2). Correcting this requires a randomized or exact test, which we have not implemented.

### 5.4. What the Theory Does and Does Not Settle

Remark 4 separates four contributions. The approximation and idealized generalization terms are partially analyzed; optimization error and design mismatch remain uncontrolled. The idealized generalization term is standard and decreases with the training budget; the approximation term, how well a small network represents the conditional oracle $\lambda^{*}$ on the FoF domain, has no rate here, and supplying one, together with the Rademacher complexity of this specific architecture, is the main problem left open. A second open question concerns what Corollary 1 does *not* provide: it characterizes when a vertex of the simplex minimizes the fixed-weight risk, but it does not establish strict dominance of the sample-conditional oracle over the per-regime one. Conditions on M(φ) under which that dominance is strict, and a quantification of the gap, remain open. Super-learner arguments [27] do not transfer directly, since weights and base estimates are functions of the same statistic.

## 6. Conclusions

We introduced ASED, a sample-conditional mixture entropy estimator for short sequences in the marginal uniformity-testing setting of symmetric cryptography. A compact MLP (6848 weight parameters), trained once offline with a regime-balanced objective, maps a 37-dimensional frequency-of-frequencies descriptor to convex weights over six classical estimators, at O(n+k) inference cost.

On the theoretical side, we prove an oracle inequality bounding the excess risk of any conditional mixture by the geometry of the conditional error matrix and the weight approximation error; a checkable covariance condition under which sample-dependent weighting is necessary for any convex-mixture improvement, which explains the regime-specific ablation findings where it holds and quantifies why a fixed mixture remains insufficient at n=32; and fixed-alphabet consistency for every simplex-valued weighting rule, independently of the training distribution, together with non-asymptotic safety bounds. The precise hypotheses of full support of the conditional oracle for the quadratic rate, and a non-degenerate asymptotic variance together with a vanishing minimax-prior weight for asymptotic normality, the former failing under the uniform null, are stated with the results in Section 3.

The evaluation proceeds in layers, each closing a specific evidentiary gap. Under the uniform-null protocol of Contreras Rodríguez et al. [2], ASED attains an integrated MSE of $1.7\times 10^{-3}$ for bytes against $3.83\times 10^{-2}$ for the best single estimator, SHR, a reduction of 95.4% under summed or averaged aggregation and 24% under the mean of per-size ratios, confined to n<k and reproduced at 95.1% under the locked confirmatory protocol of Section 4.10.3 with a paired bootstrap interval for the integrated-MSE difference excluding zero, and outperforms the per-regime best-single-estimator benchmark for n≤32; for bits, it improves on SHR by 31%, ranking second behind the minimax-prior estimator, a single-alphabet figure we do not advance as a claim for the method. MIN belongs to ASED’s own base set, and the run-to-run spread at k=2 is comparable to the margins separating the estimators. Against near-uniform alternatives of known entropy, ASED’s detection power was non-inferior to SHR’s within a post hoc margin of 0.02 at every tested operating point, pointwise and simultaneously, with one exclusion: at n≤16, the ASED and SHR statistics are monotone relabelings of one another on the null support, and no test of size 0.05 exists, so no power difference is identifiable and the comparison is withdrawn there (Section 4.5), while the constant control, which achieves zero error by construction under the null, has zero power separating genuine adaptive estimation from calibration artifacts. On held-out sample sizes and an unseen alphabet, the advantage persists.

On strongly non-uniform or periodic sources, the ordering reverses, and general-purpose estimators such as NSB dominate by an order of magnitude, a boundary we quantify rather than elide. The contribution is therefore best stated without reference to the network or to the 37-dimensional descriptor, neither of which is load-bearing: it is the sample-conditional calibration of convex mixtures of classical entropy estimators using occupancy statistics. The neural mapper and the extended descriptor are one convenient realization: a five-feature MLP and, separately, a gradient-boosted surrogate each achieved comparable performance, the two ablations having been run independently. For uniformity diagnostics on short cryptographic byte sequences, these results justify ASED as a candidate for prospective validation against SHR in short-sample marginal-uniformity diagnostics: a nested bootstrap supported pointwise and simultaneous non-inferiority over the nine pre-defined small-sample cells at the post hoc 0.02 margin, estimation error under the null is substantially lower, and the computational overhead is negligible. That prospective validation matters concretely: the 95.4% integrated-MSE reduction falls to 24% under a per-size-ratio aggregation (Section 4.4), and the feature map, architecture, and loss weighting were all selected while observing this same evaluation protocol, so a locked train/validate/test split, rather than a restatement of the present results, is what “prospective” is intended to require here. The agenda this work opens is the generalization half of the theory, a bound on $E∥\lambda -\lambda^{*}{∥}_{1}$ for the offline fit, together with base sets extended by general-purpose members such as NSB, descriptors informative at small alphabets, and a faithful comparison against BUB and Unseen.

## Figures and Tables

**Figure 1:**
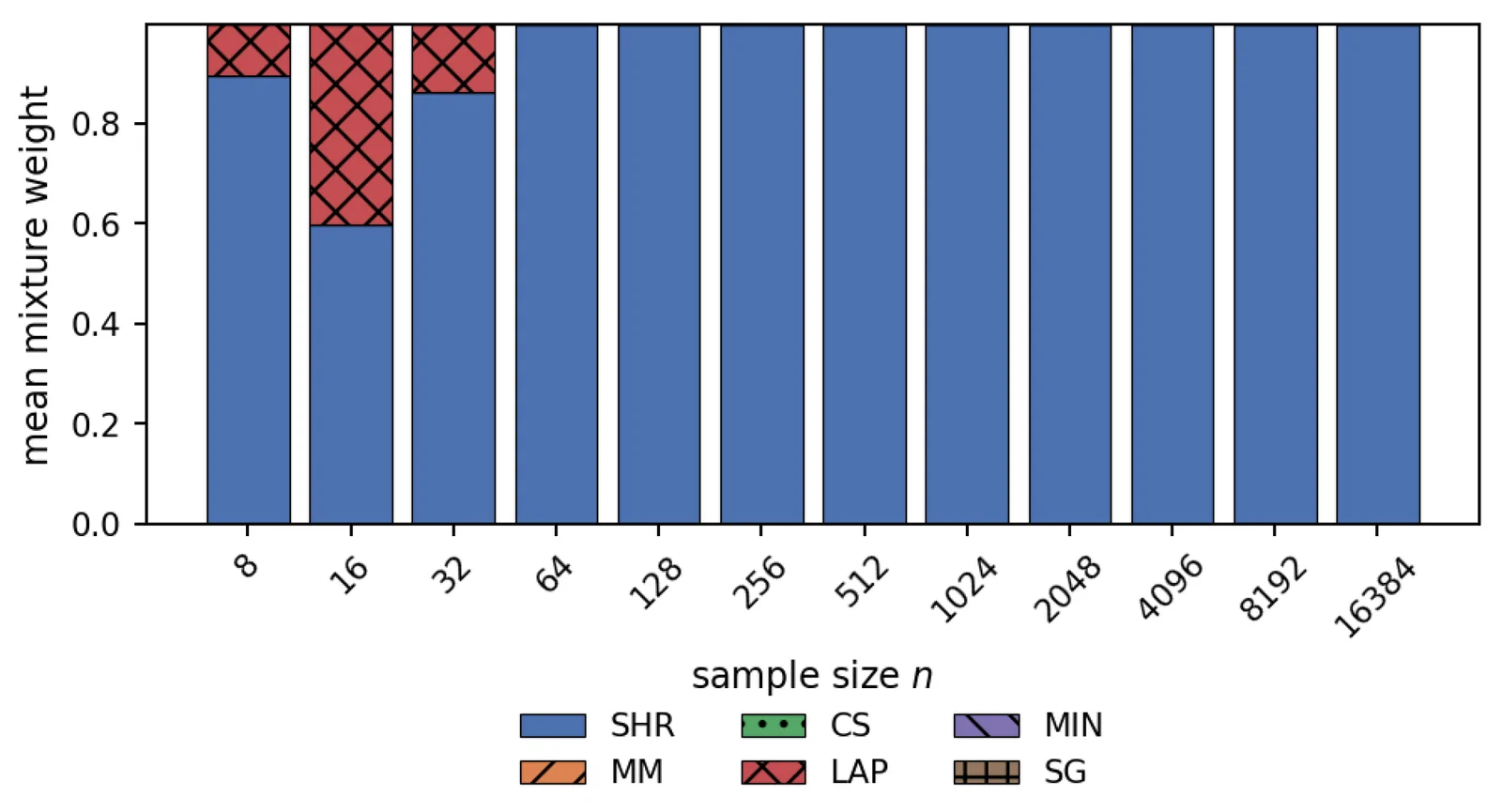
Mean adaptive mixture weights $\lambda_{j}\left(\varphi ;\theta \right)$ for byte sequences, by sample size.

**Figure 2:**
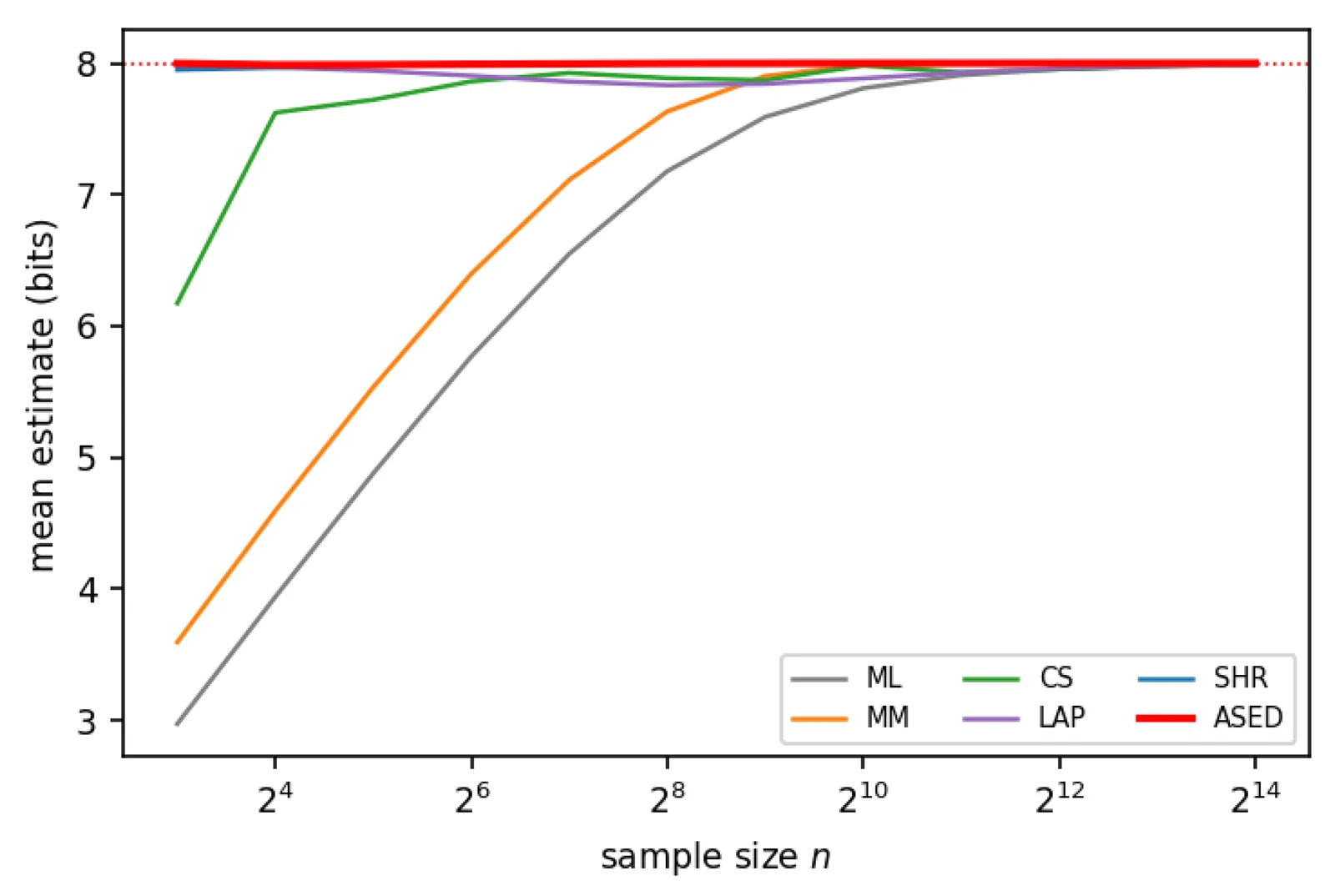
Mean entropy estimate versus sample size for byte sequences (k=256, $H_{\max}=8$, dotted line). ASED in red.

**Figure 3:**
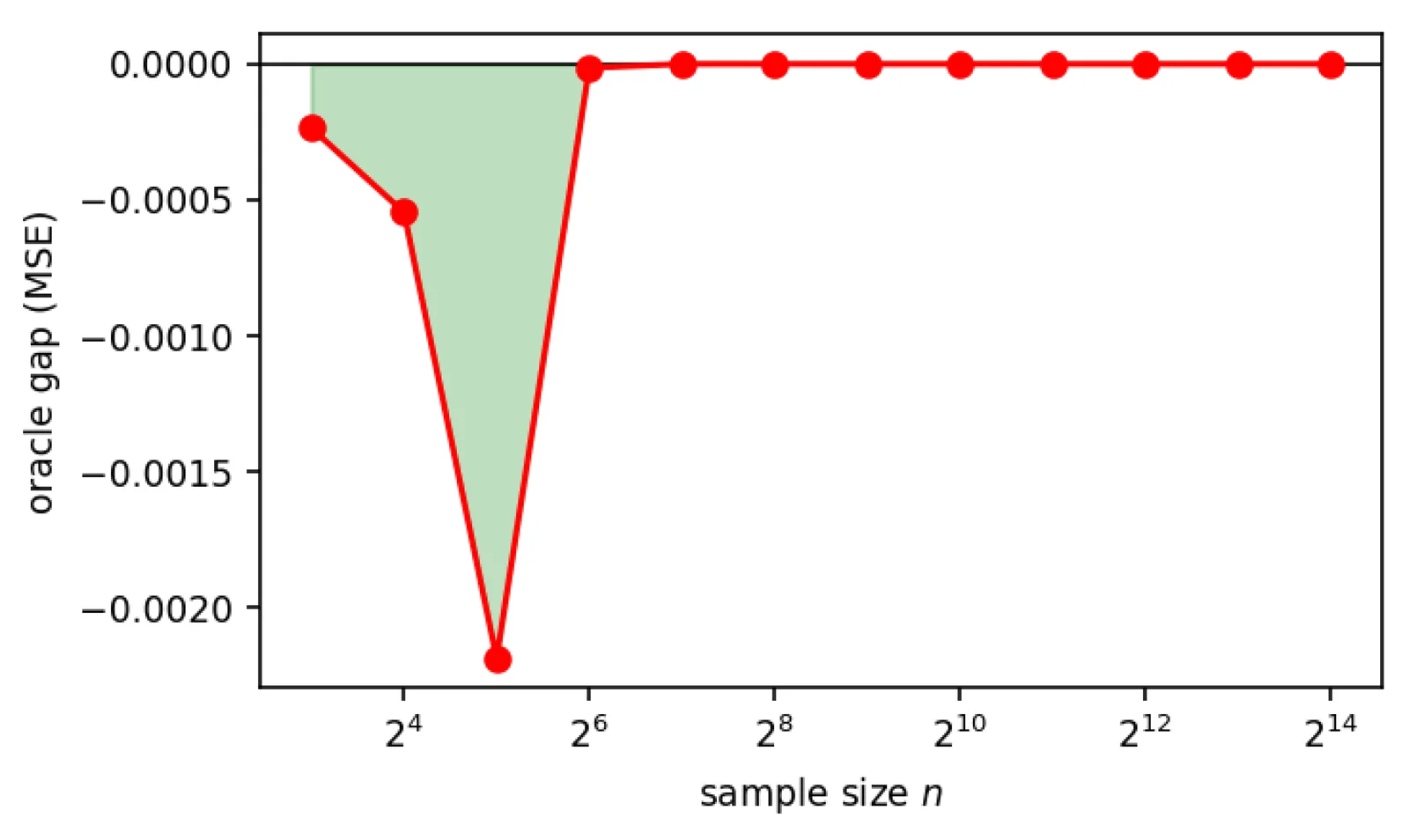
Benchmark gap $\mathrm{MSE}\left({\hat{H}}_{\mathrm{ASED}}\right)-\min_{j}\mathrm{MSE}\left({\hat{H}}_{j}\right)$ over the eight fixed estimators, per sample size. Green: ASED beats the per-regime best-single-estimator benchmark; the gap vanishes for n≥64.

**Figure 4:**
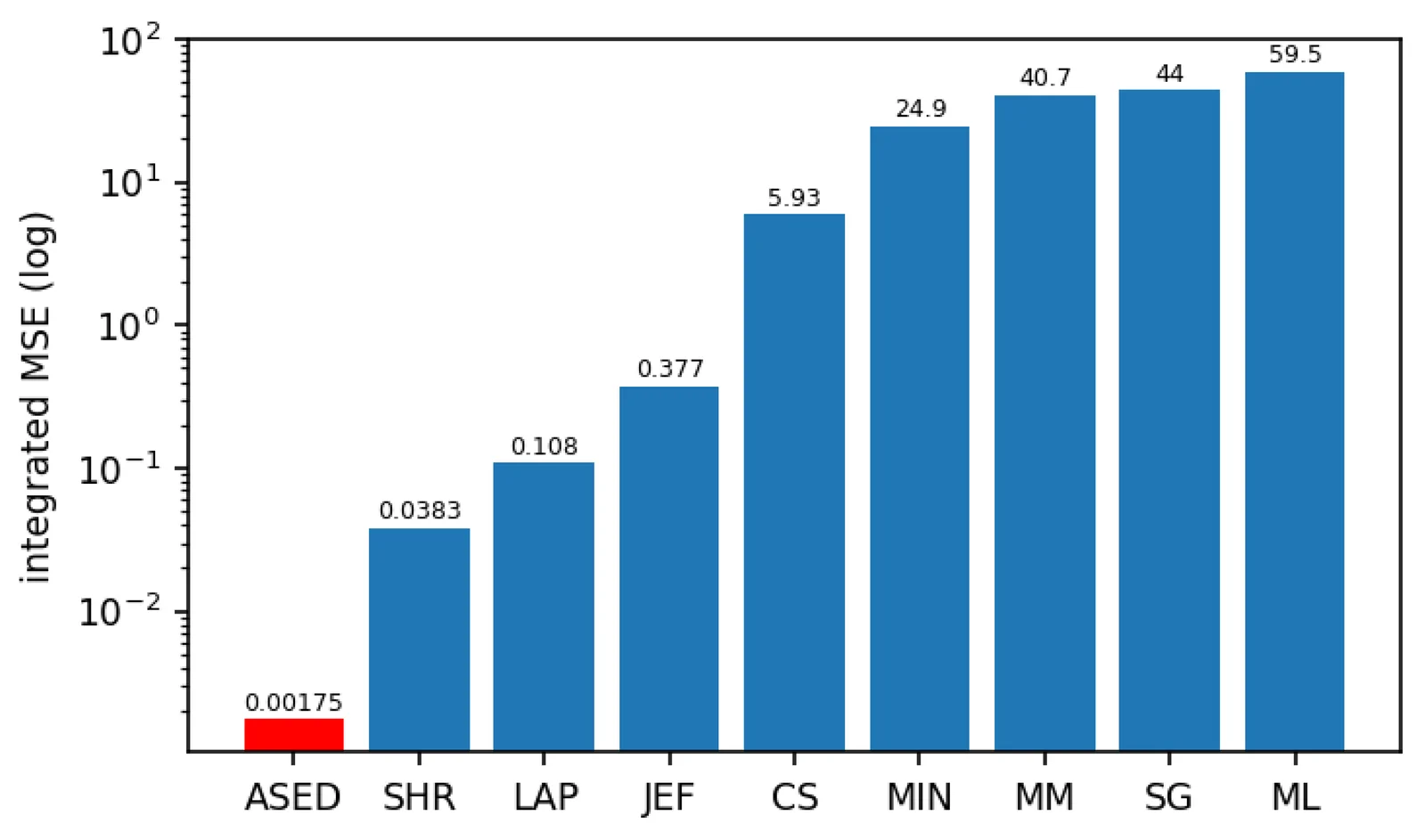
Integrated MSE for byte sequences (log scale). ASED in red.

**Figure 5:**
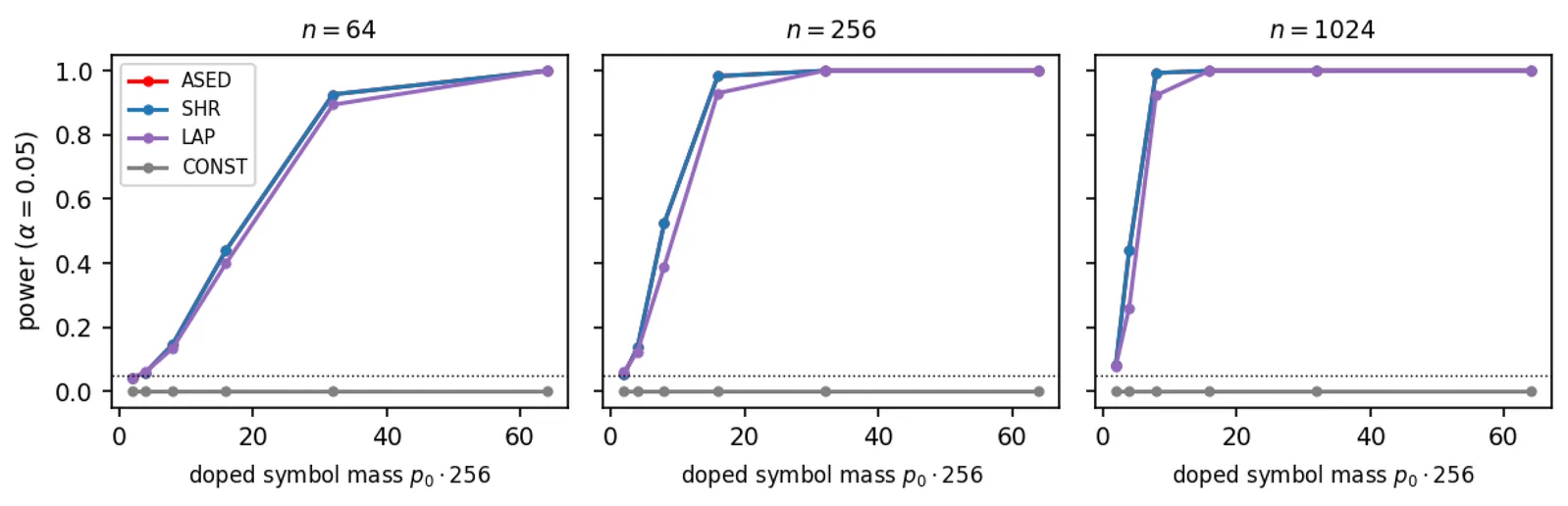
Power curves for doped-byte alternatives at n∈{64,256,1024}, α=0.05 (dotted line). The constant control (grey) has zero power throughout.

**Figure 6:**
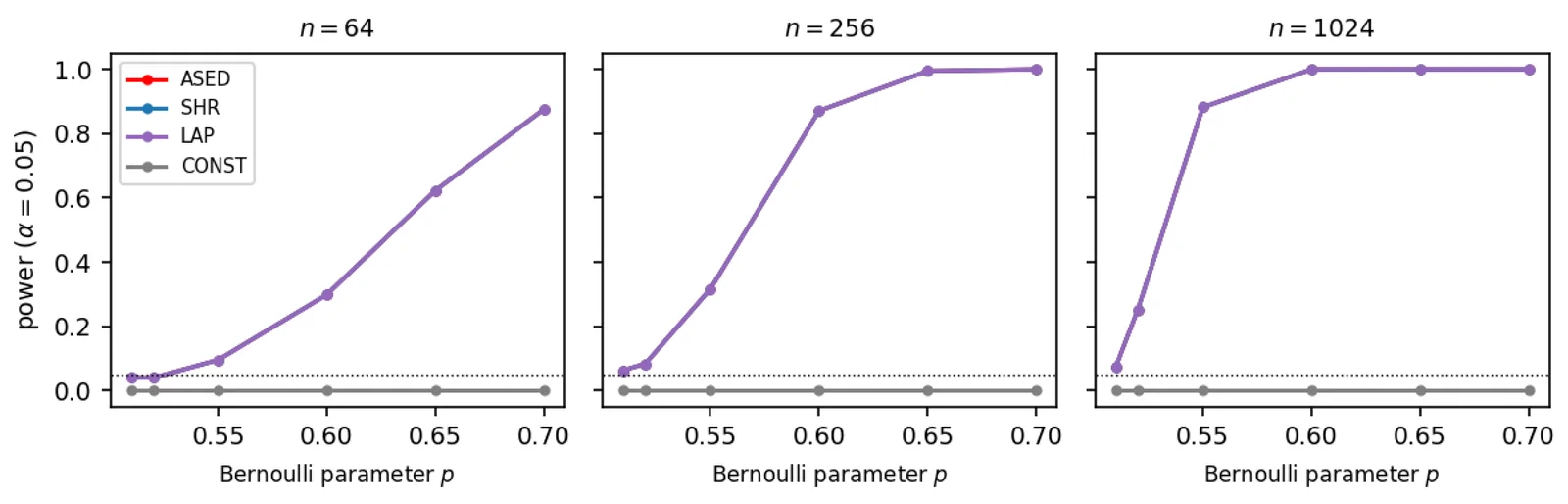
Power curves for biased-bit alternatives. All genuine estimators coincide (monotone functions of the count imbalance).

**Table 1:** ASED architecture and training hyperparameters.

Element	Value
Input dimension	37 (Equation (3))
Hidden layers/width/activation	2/64/ReLU
Output	softmax over M=6 weights
Feature preprocessing	standardization (training μ,ς)
Optimizer/learning rate	Adam/$10^{-3}$, cosine annealing
Epochs/batch size	600/256
Loss	regime-balanced MSE (Equation (5))
Entropy bonus $\beta_{t}$	linear decay 1→0 over first 30%
Training alphabets/sizes	k∈{2,256}/12 values of *n*
Replications per configuration	1000

**Table 2:** Verification of condition (8) for ${\bar{M}}_{k,n}$ of Equation (9): ratios ${\bar{M}}_{ij^{*}}/{\bar{M}}_{j^{*}j^{*}}$ for all six base estimators, k=256, from 4000 replications. The entry $i=j^{*}$ is one by construction; the condition requires every off-diagonal ratio to be at least 1.

*n*	$j^{*}$	SHR	MM	CS	LAP	MIN	SG
8	LAP	3.77	266.30	111.71	1.00	178.24	241.56
16	LAP	1.07	107.90	12.86	1.00	82.88	111.86
32	SHR	1.00	21.30	8.70	0.48	18.26	24.03
64	SHR	1.00	28.24	8.87	1.84	27.84	35.66

**Table 3:** Absolute bias $\left|\mathrm{Bias}|=\right|H_{\max}-\hat{H}|$ for byte samples (k=256, $H_{\max}=8$ bits). Best value per column in **bold**.

Est.	n=8	n=32	n=64	n=128	n=256	n=512	n=1024
ML	5.027	3.121	2.232	1.451	0.822	0.409	0.191
SHR	0.050	0.020	**0.008**	**0.005**	**0.002**	**0.001**	**0.001**
LAP	0.017	0.057	0.096	0.141	0.168	0.158	0.116
MM	4.405	2.465	1.603	0.889	0.368	0.098	0.014
ASED	**0.002**	**0.012**	**0.008**	**0.005**	**0.002**	**0.001**	**0.001**

**Table 4:** MSE for byte samples (k=256), scientific notation. Integrated MSE over all 12 sample sizes, with bootstrap 95% CIs for ASED and SHR. CONST is a degenerate control, not an estimator.

Est.	n=8	n=32	n=64	n=128	n=256	n=512	Int.
ML	$2.5\;\times \;10^{1}$	$9.8\;\times \;10^{0}$	$5.0\;\times \;10^{0}$	$2.1\;\times \;10^{0}$	$6.8\;\times \;10^{-1}$	$1.7\;\times \;10^{-1}$	$5.95\;\times \;10^{1}$
SHR	$2.7\;\times \;10^{-2}$	$2.8\;\times \;10^{-3}$	$4.2\;\times \;10^{-4}$	$1.7\;\times \;10^{-4}$	$2.8\;\times \;10^{-5}$	$1.1\;\times \;10^{-5}$	$3.83\;\times \;10^{-2}$
LAP	$2.7\;\times \;10^{-4}$	$3.3\;\times \;10^{-3}$	$9.2\;\times \;10^{-3}$	$2.0\;\times \;10^{-2}$	$2.8\;\times \;10^{-2}$	$2.5\;\times \;10^{-2}$	$1.08\;\times \;10^{-1}$
NSB	$7.7\;\times \;10^{0}$	$7.4\;\times \;10^{-1}$	$1.9\;\times \;10^{-1}$	$5.5\;\times \;10^{-2}$	$1.2\;\times \;10^{-2}$	$2.7\;\times \;10^{-3}$	$1.12\;\times \;10^{1}$
JEF	$2.8\;\times \;10^{-3}$	$2.8\;\times \;10^{-2}$	$6.2\;\times \;10^{-2}$	$9.7\;\times \;10^{-2}$	$9.3\;\times \;10^{-2}$	$5.5\;\times \;10^{-2}$	$3.77\;\times \;10^{-1}$
ASED	$3.8\;\times \;10^{-5}$	$6.5\;\times \;10^{-4}$	$4.0\;\times \;10^{-4}$	$1.7\;\times \;10^{-4}$	$2.8\;\times \;10^{-5}$	$1.1\;\times \;10^{-5}$	$1.75\;\times \;10^{-3}$
CONST	0	0	0	0	0	0	0

**Table 5:** Integrated MSE ranking for bit sequences (k=2), from a single locked evaluation with 4000 replications per sample size and percentile bootstrap 95% intervals. This is the locked reference for the main bit-sequence comparison; the auxiliary protocols reported in Section 4.6 and Section 4.8 should not be read as replications of it. SG and JEF are tied at rank 6 to the reported precision (both 0.0231) and share that rank rather than being assigned ranks 6 and 7.

Rank	Estimator	Int. MSE	Bootstrap 95% CI
1	MIN	0.0109	[0.0102,0.0116]
2	ASED	0.0135	[0.0122,0.0147]
3	LAP	0.0158	[0.0148,0.0168]
4	SHR	0.0196	[0.0166,0.0227]
5	CS	0.0215	[0.0187,0.0245]
6	SG	0.0231	[0.0215,0.0248]
6	JEF	0.0231	[0.0215,0.0248]
8	MM	0.0270	[0.0240,0.0302]
9	ML	0.0376	[0.0343,0.0409]

**Table 6:** Detection power at α=0.05 for doped-byte alternatives, n=256. CONST is the constant control. Empirical size at the null is ≈0.05 for all genuine estimators.

$p_{0}$	*H* (bits)	ASED	SHR	LAP	CONST
2/256	7.998	0.053	0.053	0.061	0.000
4/256	7.986	0.137	0.137	0.124	0.000
8/256	7.945	0.524	0.524	0.389	0.000
16/256	7.832	0.983	0.983	0.930	0.000
32/256	7.539	1.000	1.000	1.000	0.000
64/256	6.807	1.000	1.000	1.000	0.000

**Table 7:** MSE against the true entropy of doped-byte alternatives, n=64 (the regime where the estimators differ).

$p_{0}$	*H* (bits)	ASED	SHR	LAP
2/256	7.998	0.0005	0.0005	0.0088
4/256	7.986	0.0006	0.0007	0.0069
8/256	7.945	0.0029	0.0039	0.0020
16/256	7.832	0.0198	0.0296	0.0039
32/256	7.539	0.1126	0.2027	0.1088
64/256	6.807	0.9676	0.8280	0.9765

**Table 8:** Detection power at α=0.05 for doped-byte alternatives at the small sample sizes, where the MSE gain is concentrated (k=256). Half-widths of 95% binomial intervals are at most ±0.022; a paired non-inferiority test at margin δ=0.02 is reported in the text.

*n*	$p_{0}$	ASED	SHR	LAP	MIN	CONST
8	32/256	0.07	0.08	0.07	0.07	0.00
	64/256	0.33	0.34	0.33	0.33	0.00
	128/256	0.86	0.86	0.86	0.86	0.00
16	16/256	0.10	0.09	0.10	0.10	0.00
	32/256	0.36	0.34	0.36	0.36	0.00
	64/256	0.81	0.81	0.81	0.81	0.00
32	16/256	0.28	0.28	0.28	0.28	0.00
	32/256	0.70	0.70	0.70	0.70	0.00
	64/256	0.99	0.99	0.99	0.99	0.00

**Table 9:** Detection power at nominal α=0.05: ASED and SHR versus a simulation-calibrated Pearson $\chi^{2}$ test and a likelihood-ratio (G) test, doped-byte alternatives, from a single paired run (4000 null and 4000 alternative replications per cell, all four detectors on the same draws). 95% Wilson intervals in brackets; best value per row in bold. The strict tie convention of Section 4.10.2 is used throughout, which is conservative at the smallest sample sizes.

*n*	$p_{0}$	$\chi^{2}$	G-Test	ASED	SHR
8	32/256	0.086 [0.078, 0.095]	0.098 [0.089, 0.108]	0.109 [0.100, 0.119]	0.091 [0.083, 0.101]
	64/256	0.337 [0.323, 0.352]	0.340 [0.326, 0.355]	0.369 [0.354, 0.384]	0.344 [0.330, 0.359]
	128/256	0.861 [0.850, 0.871]	0.862 [0.851, 0.872]	0.872 [0.862, 0.882]	0.862 [0.851, 0.872]
16	16/256	0.082 [0.074, 0.091]	0.082 [0.074, 0.091]	0.082 [0.074, 0.091]	0.140 [0.129, 0.151]
	32/256	0.346 [0.332, 0.361]	0.346 [0.332, 0.361]	0.346 [0.332, 0.361]	0.404 [0.388, 0.419]
	64/256	0.796 [0.783, 0.808]	0.796 [0.783, 0.808]	0.796 [0.784, 0.809]	0.822 [0.809, 0.833]
32	16/256	0.193 [0.181, 0.206]	0.248 [0.235, 0.261]	0.220 [0.207, 0.233]	0.259 [0.245, 0.272]
	32/256	0.630 [0.615, 0.645]	0.640 [0.625, 0.655]	0.648 [0.633, 0.663]	0.678 [0.664, 0.693]
	64/256	0.980 [0.975, 0.984]	0.977 [0.972, 0.981]	0.982 [0.977, 0.985]	0.984 [0.980, 0.987]

**Table 10:** Generalization: integrated MSE on configurations excluded from training.

Configuration	ASED	SHR	LAP
Held-out sizes *n* (bytes)	0.0014	0.0075	0.0541
In-grid sizes *n* (bytes)	0.0008	0.0252	0.0546
Unseen alphabet k=16 (full grid)	0.0195	0.0337	0.0938

**Table 11:** Interpolation across alphabets: integrated MSE on held-out alphabets k∈{32,128} and on the training alphabets, for a mapper trained on k∈{2,16,64,256} versus the main model trained on k∈{2,256}.

Alphabet	ASED (Trained on 4 Alphabets)	ASED (Trained on {2,256})	SHR
k=32 (held out)	0.0092	0.0106	0.0290
k=128 (held out)	0.0035	0.0036	0.0319
k=256	0.0018	0.0018	0.0384
k=2	0.0092	0.0074	0.0092

**Table 12:** Ablation on the feature map (bytes, integrated MSE, common evaluation stream, 500 replications per size). SHR on the same stream: 0.0327.

Model	Features	Int. MSE
Fixed global mixture	0	0.0324
n/k-only mapper	1	0.0320
Regime-summary mapper	5	0.0017
Full ASED	37	0.0017

**Table 13:** Integrated MSE including general-purpose estimators, common evaluation stream (200 replications per sample size). Absolute values differ slightly from Table 4 because of the smaller replication count; the ranking is the object of interest.

Estimator	Bytes (k=256)	Bits (k=2)
ASED	0.0016	0.0217
SHR	0.0298	0.0504
LAP	0.1082	0.0247
MIN	24.81	0.0170
CS	5.958	0.0543
CWJ	9.275	0.0573
NSB	11.165	0.0843
GR	19.039	0.1205
MM	40.534	0.0576
SG	43.932	0.0379

**Table 14:** MSE against the true (marginal) entropy for structured sources, k=256, n=256, 400 replications.

Source	*H* (bits)	ASED	SHR	LAP	NSB
Zipf s=0.3	7.916	**0.005**	**0.005**	0.011	0.010
Zipf s=0.6	7.531	0.029	0.026	0.034	**0.018**
Zipf s=1.0	6.222	1.173	0.073	1.179	**0.040**
Markov $p_{\mathrm{stay}}\;=\;0.5$	8.000	**0.154**	0.469	**0.154**	1.381
Markov $p_{\mathrm{stay}}\;=\;0.9$	8.000	1.412	11.821	**1.346**	14.507
Periodic (period 64)	6.000	2.399	1.436	2.399	**0.065**

**Table 15:** Weight-function families under the identical protocol (bytes, integrated MSE). Direct: fitted against Equation (5). Surrogate: per-estimator squared-error regression, converted to weights by softmax or by selecting the predicted minimum.

Family	Strategy	Int. MSE
Multinomial logistic (no hidden layer)	direct	$7.6\times 10^{-3}$
Additive spline model (GAM-like)	direct	$2.0\times 10^{-3}$
MLP, one hidden layer of width 16	direct	$1.86\times 10^{-3}$
MLP, two hidden layers of width 64 (ASED)	direct	$1.71\times 10^{-3}$
Nadaraya–Watson kernel regression	surrogate	$1.05\times 10^{-2}$
Random forest	surrogate (softmax)	$2.30\times 10^{-3}$
Random forest	surrogate (select)	$2.01\times 10^{-3}$
Gradient boosting	surrogate (softmax)	$3.04\times 10^{-3}$
Gradient boosting	surrogate (select)	$1.54\times 10^{-3}$
Binned lookup on (n/k,C)	surrogate (select)	$2.14\times 10^{-3}$
SHR (best fixed estimator)		$3.83\times 10^{-2}$

**Table 16:** Paired comparison of three weight-function families on one locked evaluation stream (independent implementation, 1000 replications per sample size, 4000 paired bootstrap resamples of the replication index). Absolute values are higher than in Table 15 because these variants were fitted under a shorter common training budget for comparability; the differences, not the levels, are the object of interest.

Comparison	Difference in Integrated MSE	95% Paired Bootstrap CI
Full mapper − ASED-lite	$+1.21\times 10^{-3}$	$\left[-1.42\times 10^{-4},\;+3.82\times 10^{-3}\right]$
Full mapper − gradient boosting	$+1.34\times 10^{-3}$	$\left[-2.93\times 10^{-5},\;+4.00\times 10^{-3}\right]$
ASED-lite − gradient boosting	$+1.28\times 10^{-4}$	$\left[-2.02\times 10^{-5},\;+3.19\times 10^{-4}\right]$

**Table 17:** Mean entropy estimate on generator output (k=256, 400 windows). “Target *H*” is the marginal entropy the source is designed to have: 8 bits for the two reference streams and for RC4 initial output when positions are pooled (its position-specific deficit is analyzed separately below) and the exact value for the biased source. The truncated LCG has an exactly uniform marginal on a support of 128 values; hence, H=7 bits (see text).

Source	Target *H*	n=32		n=128		n=512	
		ASED	SHR	ASED	SHR	ASED	SHR
SHA-256 counter mode	8 (design)	7.989	7.986	7.995	7.995	7.998	7.998
RC4, first 768 bytes dropped	8 (design)	7.988	7.983	7.995	7.995	7.999	7.999
RC4, initial output	≈8 (pooled)	7.989	7.986	7.995	7.995	7.999	7.999
Truncated LCG	7 (exact)	7.962	7.914	7.826	7.804	7.554	7.553
Biased source $p_{0}=8/256$	7.945 (exact)	7.982	7.964	7.972	7.972	7.959	7.959

**Table 18:** RC4 $Z_{2}$ detection: rejection rate at α=0.05, ASED and SHR (entropy-based, calibrated by simulation) versus a direct test on the count of $Z_{2}=0$ occurrences (calibrated against the exact null binomial). 95% Wilson intervals in brackets.

*n*	ASED	SHR	Direct $\mathrm{Pr}\{Z_{2}=0\}$ Test
500	0.061 [0.051, 0.072]	0.061 [0.051, 0.072]	0.425 [0.404, 0.447]
2000	0.149 [0.134, 0.165]	0.149 [0.135, 0.166]	0.771 [0.753, 0.789]
5000	0.393 [0.363, 0.424]	0.425 [0.395, 0.456]	0.983 [0.973, 0.989]
20,000	0.993 [0.978, 0.997]	0.993 [0.978, 0.997]	1.000 [0.990, 1.000]
50,000	1.000 [0.981, 1.000]	1.000 [0.981, 1.000]	1.000 [0.981, 1.000]

**Table 19:** Discreteness of the ASED null distribution and the resulting sensitivity of reported power to the tie convention (k=256, nominal α=0.05, 8000 null and 4000 alternative replications). “Mass at crit” is the null probability exactly at the empirical critical value. “Gap” is the change in reported power from the tie rule alone.

*n*	Distinct Null Values	Mass at Crit.	Power (Strict)	Power (Inclusive)	Gap
8	4	0.100	0.374	0.655	0.281
16	9	0.058	0.341	0.435	0.094
32	23	0.055	0.692	0.717	0.025
64	89	0.012	0.473	0.482	0.010
128	600	0.000	0.289	0.289	0.000

**Table 20:** Independent verification, byte sequences (k=256): per-size MSE from a separate implementation with a locked train/validate/test split (three independent random-seed streams, test evaluated once). The per-size column sums to the integrated total exactly, and the n<k subset ($1.694\times 10^{-3}$ for ASED, $3.559\times 10^{-2}$ for SHR) is strictly below it, as it must be.

*n*	ASED	SHR	Reduction
8	$3.98\;\times \;10^{-5}$	$2.60\;\times \;10^{-2}$	99.8%
16	$4.17\;\times \;10^{-4}$	$6.94\;\times \;10^{-3}$	94.0%
32	$5.85\;\times \;10^{-4}$	$1.84\;\times \;10^{-3}$	68.3%
64	$4.84\;\times \;10^{-4}$	$6.20\;\times \;10^{-4}$	21.9%
128	$1.68\;\times \;10^{-4}$	$1.67\;\times \;10^{-4}$	−0.5%
256	$3.38\;\times \;10^{-5}$	$3.38\;\times \;10^{-5}$	−0.1%
512	$1.29\;\times \;10^{-5}$	$1.29\;\times \;10^{-5}$	−0.1%
1024	$3.05\;\times \;10^{-6}$	$2.91\;\times \;10^{-6}$	−4.5%
2048	$5.86\;\times \;10^{-7}$	$5.84\;\times \;10^{-7}$	−0.3%
4096	$1.12\;\times \;10^{-7}$	$1.12\;\times \;10^{-7}$	−0.0%
8192	$3.37\;\times \;10^{-8}$	$3.37\;\times \;10^{-8}$	−0.1%
16384	$7.08\;\times \;10^{-9}$	$7.08\;\times \;10^{-9}$	−0.1%
Integrated	$1.744\;\times \;10^{-3}$	$3.564\;\times \;10^{-2}$	95.1%

**Table 21:** Internally consistent single-run regeneration of the byte-sequence evaluation, from the independent implementation of Section 4.10.3 (seed fixed, 1000 replications per size, disjoint regimes). All aggregates below the rule are computed from the per-size column above it.

*n*	ASED	SHR	Reduction
8	$3.73\;\times \;10^{-5}$	$2.63\;\times \;10^{-2}$	99.9%
16	$4.41\;\times \;10^{-4}$	$6.43\;\times \;10^{-3}$	93.1%
32	$5.85\;\times \;10^{-4}$	$2.39\;\times \;10^{-3}$	75.5%
64	$5.61\;\times \;10^{-4}$	$6.44\;\times \;10^{-4}$	12.8%
128	$1.46\;\times \;10^{-4}$	$1.46\;\times \;10^{-4}$	−0.2%
256	$3.63\;\times \;10^{-5}$	$3.62\;\times \;10^{-5}$	−0.1%
512	$9.70\;\times \;10^{-6}$	$9.69\;\times \;10^{-6}$	−0.1%
1024	$2.44\;\times \;10^{-6}$	$2.38\;\times \;10^{-6}$	−2.6%
2048	$5.55\;\times \;10^{-7}$	$5.54\;\times \;10^{-7}$	−0.1%
4096	$1.29\;\times \;10^{-7}$	$1.28\;\times \;10^{-7}$	−0.3%
8192	$3.74\;\times \;10^{-8}$	$3.73\;\times \;10^{-8}$	−0.2%
16,384	$9.90\;\times \;10^{-9}$	$9.90\;\times \;10^{-9}$	−0.0%
Integrated	$1.819\;\times \;10^{-3}$	$3.598\;\times \;10^{-2}$	94.94%
Disjoint regime sums (add to the integrated total exactly):			
n<k	$1.770\;\times \;10^{-3}$	$3.593\;\times \;10^{-2}$	95.1%
k≤n≤512	$4.596\;\times \;10^{-5}$	$4.592\;\times \;10^{-5}$	−0.1%
n>512	$3.174\;\times \;10^{-6}$	$3.112\;\times \;10^{-6}$	−2.0%

## Data Availability

The complete implementation is released in support of the results reported here: the reference implementation of ASED and of all base estimators; the trained weight-mapper coefficients together with the feature-standardization constants μ and ς belonging to each trained model, including every retrained variant of Section 4.6, Section 4.9 and Section 4.10.3; the independently written implementation used for the locked confirmatory evaluation; the random seeds and seed-stream definitions of every experiment; and the evaluation and aggregation scripts that regenerate each table and figure in this paper from those seeds. In view of the inconsistency documented in Section 4.11, the aggregation scripts carry the internal-consistency conditions as run-time assertions, so that regime sums, subset sums, and paired differences are checked against their own per-size constituents whenever a table is produced. The archive is deposited in a public repository under a permanent identifier, to be inserted at proof stage; until then, it is available from the corresponding author on request.

## Supplementary Materials

The following supporting information can be downloaded at: https://www.mdpi.com/article/10.3390/e28101073/s1. Code S1: reference implementation of ASED and of the six base estimators, the weight-mapper trainer, the locked evaluation harness with its three disjoint random-seed streams, and the aggregation module in which the internal-consistency conditions of Section 4.11 are enforced as run-time assertions; a regression test suite covering each of those conditions is included, and run_all.py regenerates the byte-sequence and bit-sequence evaluations end to end in under a minute per configuration on one CPU core. Data S1: trained weight-mapper coefficients (the 6848 weights and 134 biases of Equation (4)) together with the feature standardization constants μ and ς, one set per trained model, since one model’s constants are not valid with another model’s weights (Section 4.10.3). Data S2: full-precision per-size results for every estimator, sample size, and configuration reported in Table 3, Table 4, Table 5, Table 6, Table 7, Table 8, Table 9, Table 10, Table 11, Table 12, Table 13, Table 14, Table 15, Table 16 and Table 17, in the disjoint regime partition used throughout. Code S2: the independent implementation used for the locked confirmatory evaluation of Section 4.10.3, together with the auxiliary scripts for the RC4 $Z_{2}$ analysis, the Zipf sweep, the support-restricted generator, and the fresh-null power calibration; it is not the pipeline behind the exploratory results and is not guaranteed to share every implementation detail with it.

## Abbreviations

The following abbreviations are used in this manuscript:

ASED	Adaptive sample-conditional entropy diagnostic
FoF	Frequency-of-frequencies
MSE	Mean squared error
SHR	James–Stein shrinkage estimator
NSB	Nemenman–Shafee–Bialek estimator
MM	Miller–Madow estimator
CS	Chao–Shen estimator

## Appendix A. Base Estimator Definitions

All estimators operate on the counts $y_{x}$, with ${\hat{p}}_{x}=y_{x}/n$, $m_{\mathrm{obs}}=\left|\left\{x:y_{x}>0\right\}\right|$, and entropies in bits.

Dirichlet family.

For a prior pseudocount a>0, the smoothed frequencies and plug-in entropy are

(A1) $${\hat{p}}_{x}^{\left(a\right)}=\frac{y_{x}+a}{n+ka},\;{\hat{H}}_{a}=-\sum_{x\in K}{\hat{p}}_{x}^{\left(a\right)}\log_{2}{\hat{p}}_{x}^{\left(a\right)},$$

with the four members used here written explicitly (note that the MIN pseudocount is $a_{n}=\sqrt{n}/k$, not $\sqrt{n/k}$, so that $ka_{n}=\sqrt{n}$ and the denominator is $n+\sqrt{n}$; the displayed frequencies sum to one exactly):

(A2) $${\hat{p}}_{x}^{\;\mathrm{LAP}}=\frac{y_{x}+1}{n+k},\;{\hat{p}}_{x}^{\;\mathrm{JEF}}=\frac{y_{x}+1/2}{n+k/2},\;{\hat{p}}_{x}^{\;\mathrm{SG}}=\frac{y_{x}+1/k}{n+1},\;{\hat{p}}_{x}^{\;\mathrm{MIN}}=\frac{y_{x}+\frac{\sqrt{n}}{k}}{n+\sqrt{n}}.$$

Plug-in, corrected, and coverage-based estimators.

(A3) $$\begin{array}{cc} {\hat{H}}_{\mathrm{ML}} & =-\sum_{x}{\hat{p}}_{x}\log_{2}{\hat{p}}_{x}, \end{array}$$

(A4) $$\begin{array}{cc} {\hat{H}}_{\mathrm{MM}} & ={\hat{H}}_{\mathrm{ML}}+\frac{m_{\mathrm{obs}}-1}{2n\ln2}, \end{array}$$

(A5) $$\begin{array}{cc} {\hat{H}}_{\mathrm{CS}} & =-\sum_{x:y_{x}>0}\frac{{\hat{p}}_{x}^{\;C}\log_{2}{\hat{p}}_{x}^{\;C}}{1-{\left(1-{\hat{p}}_{x}^{\;C}\right)}^{n}},\;{\hat{p}}_{x}^{\;C}=C\;{\hat{p}}_{x},\;C=1-\frac{f_{1}}{n}, \end{array}$$

with the guard C←1/n when $f_{1}=n$.

Shrinkage estimator.

SHR [22] plugs in the convex combination with the uniform target,

(A6) $${\hat{p}}_{x}^{\;\mathrm{SHR}}={\hat{\lambda }}^{*}\;\frac{1}{k}+\left(1-{\hat{\lambda }}^{*}\right)\;{\hat{p}}_{x},\;{\hat{\lambda }}^{*}=\min\;\left\{1,\;\max\;\left[0,\;\frac{1-\sum_{x}{\hat{p}}_{x}^{\;2}}{\left(n-1\right)\sum_{x}{\left(1/k-{\hat{p}}_{x}\right)}^{2}}\right]\right\},$$

i.e., the data-driven shrinkage intensity toward the uniform target with explicit clipping to [0,1]. If the denominator vanishes (the empirical distribution equals the uniform target), we set ${\hat{\lambda }}^{*}=1$; for n≤1, the uniform plug-in is returned directly.

## Appendix B. Uniform-Convergence Bound for the Offline Fit

**Proposition** **A1** (PAC bound for an idealized empirical-risk minimizer)**.** *Assume (H1) the training pairs $\left(\varphi_{i},e_{i}\right)$, i≤N, are i.i.d. draws from the training distribution; (H2) for the idealized procedure considered only in this proposition, base estimates are clipped to [0,B] with $B\geq \log_{2}k$, so that both the clipped estimates and the true entropy lie in [0,B] and the loss $\ell \left(\lambda ;\varphi ,e\right)={\left(\sum_{j}\lambda_{j}{\hat{H}}_{j}-H\right)}^{2}$ takes values in $\left[0,B^{2}\right]$; and (H3) $\hat{\lambda }$ minimizes the empirical risk $\hat{R}$ over a class* Λ *of weight functions with values in $\Delta^{M}$,* specified before seeing the data *(for instance, by norm constraints fixed in advance). Then, for any δ∈(0,1), with probability at least 1−δ over the training sample,*

(A7) $$R\left(\hat{\lambda }\right)\;\leq \;\underset{\lambda \in \Lambda }{\inf}R\left(\lambda \right)\;+\;8B\;R_{N}\left(F_{\Lambda }\right)\;+\;2B^{2}\sqrt{\frac{2\log(2/\delta )}{N}},$$

*where $F_{\Lambda }=\left\{\left(\varphi ,e\right)\mapsto \sum_{j}\lambda_{j}\left(\varphi \right){\hat{H}}_{j}:\lambda \in \Lambda \right\}$, and $R_{N}$ is the Rademacher complexity with respect to the training distribution.*

**Remark** **A1** (What this does and does not describe)**.** *Proposition A1 bounds an idealized procedure, not the training actually run in Section 3, which differs in four respects: sampling is stratified with exactly 1000 replications per configuration rather than i.i.d.; the loss is regime-weighted and carries an epoch-dependent entropy term; the objective is non-convex and optimized by Adam, so no global minimizer is certified; and the class is not norm-constrained a priori. The honest form of the guarantee for the deployed model is therefore*

(A8) $$R\left(\hat{\lambda }\right)\;\leq \;\underset{\lambda \in \Lambda }{\inf}R\left(\lambda \right)\;+\;\underset{\mathrm{Prop}.\;A1\;}{\underset{︸}{\mathrm{generalization}\;\mathrm{error}}}\;+\;\underset{\mathrm{uncontrolled}}{\underset{︸}{\mathrm{optimization}\;\mathrm{error}}}\;+\;\underset{\mathrm{stratification},\;\mathrm{weighted}\;\mathrm{loss}}{\underset{︸}{\mathrm{design}\;\mathrm{mismatch}}},$$

*and adapting the concentration step to a stratified design with a weighted loss, together with bounding the optimization error, is left open. For the same reason the norm-based evaluation below, which uses the norms of the* trained *weights, describes a data-dependent class and would require a localization, peeling, or sample-splitting argument to be a valid uniform bound; we report it as a numerical illustration only.*

**Proof.** Write $\Delta_{N}=\sup_{\lambda \in \Lambda }\left(R\left(\lambda \right)-\hat{R}\left(\lambda \right)\right)$. By (H2), the loss lies in $\left[0,B^{2}\right]$, so changing one training pair changes $\Delta_{N}$ by at most $B^{2}/N$; McDiarmid’s inequality gives $\Delta_{N}\leq E\left[\Delta_{N}\right]+B^{2}\sqrt{\log\left(1/\delta^{\prime }\right)/\left(2N\right)}$ with probability $1-\delta^{\prime }$, and by (H1), standard symmetrization gives $E\left[\Delta_{N}\right]\leq 2R_{N}\left(\ell \circ \Lambda \right)$. The map $u\mapsto {\left(u-H\right)}^{2}$ is 2B-Lipschitz on the range of $F_{\Lambda }$ because |u−H|≤B there, so Talagrand’s contraction principle yields $R_{N}\left(\ell \circ \Lambda \right)\leq 2B\;R_{N}\left(F_{\Lambda }\right)$. Hence, $R\left(\hat{\lambda }\right)\leq \hat{R}\left(\hat{\lambda }\right)+4BR_{N}\left(F_{\Lambda }\right)+B^{2}\sqrt{\log\left(1/\delta^{\prime }\right)/\left(2N\right)}$. Let $\lambda^{\Lambda }$ attain $\inf_{\Lambda }R$; by (H3), $\hat{R}\left(\hat{\lambda }\right)\leq \hat{R}\left(\lambda^{\Lambda }\right)$, and a second application of the same concentration argument to the single function $\lambda^{\Lambda }$ (or to the supremum, as here) bounds $\hat{R}\left(\lambda^{\Lambda }\right)\leq R\left(\lambda^{\Lambda }\right)+4BR_{N}\left(F_{\Lambda }\right)+B^{2}\sqrt{\log\left(1/\delta^{\prime }\right)/\left(2N\right)}$. Combining the two displays with $\delta^{\prime }=\delta /2$ gives (A7). □

**Remark** **A2** (Evaluating the complexity term)**.** *For the architecture of Equation (4), $R_{N}\left(F_{\Lambda }\right)$ admits an explicit norm-based bound: composing the depth-3 ReLU bound of Golowich et al. [36] with the vector-contraction inequality of Maurer [37] for the softmax layer gives $R_{N}\left(F_{\Lambda }\right)≲B\;\rho \;\prod_{l}{∥W_{l}∥}_{F}\;\left(1+\sqrt{2L\log2}\right)/\sqrt{N}$, with $\rho =\max_{i}{∥z_{i}∥}_{2}$. Evaluated at the trained weights ($∥W_{1}{∥}_{F}=11.3$, $∥W_{2}{∥}_{F}=16.5$, $∥W_{3}{∥}_{F}=5.8$, ρ=41.3, N=24,000), this yields a value of order $10^{3}$: the bound is numerically vacuous at our training size, as norm-based bounds for networks typically are. Moreover, these are the norms of the* trained *weights, so the class is data-dependent, and the evaluation is not a valid uniform bound; we report it only to make the constants and the $N^{-1/2}$ scaling explicit. A direct Monte Carlo estimate of the empirical Rademacher complexity of $F_{\Lambda }$ maximizing $N^{-1}\sum_{i}\sigma_{i}f\left(\varphi_{i},e_{i}\right)$ by gradient ascent over five independent sign draws on a* 5000*-sample subset and gives 0.056±0.059, three to four orders of magnitude below the norm-based bound. Since local optimization attains the supremum only approximately, this is an estimate rather than a certificate; closing the gap between the two numbers is precisely the open problem noted in Remark 4.*
